# Expression of Alphavirus Nonstructural Protein 2 (nsP2) in Mosquito Cells Inhibits Viral RNA Replication in Both a Protease Activity-Dependent and -Independent Manner

**DOI:** 10.3390/v14061327

**Published:** 2022-06-17

**Authors:** Liubov Cherkashchenko, Kai Rausalu, Sanjay Basu, Luke Alphey, Andres Merits

**Affiliations:** 1 Institute of Technology, University of Tartu, Nooruse 1, 50411 Tartu, Estonia; liubov.cherkashchenko@ut.ee (L.C.); kai.rausalu@ut.ee (K.R.); 2 The Pirbright Institute, Ash Road, Pirbright GU24 ONF, UK; sanjay.basu@oxitec.com (S.B.); luke.alphey@pirbright.ac.uk (L.A.)

**Keywords:** alphaviruses, nsP2, protease, RNA replication, mosquito, superinfection exclusion

## Abstract

Alphaviruses are positive-strand RNA viruses, mostly being mosquito-transmitted. Cells infected by an alphavirus become resistant to superinfection due to a block that occurs at the level of RNA replication. Alphavirus replication proteins, called nsP1-4, are produced from nonstructural polyprotein precursors, processed by the protease activity of nsP2. Trans-replicase systems and replicon vectors were used to study effects of nsP2 of chikungunya virus and Sindbis virus on alphavirus RNA replication in mosquito cells. Co-expressed wild-type nsP2 reduced RNA replicase activity of homologous virus; this effect was reduced but typically not abolished by mutation in the protease active site of nsP2. Mutations in the replicase polyprotein that blocked its cleavage by nsP2 reduced the negative effect of nsP2 co-expression, confirming that nsP2-mediated inhibition of RNA replicase activity is largely due to nsP2-mediated processing of the nonstructural polyprotein. Co-expression of nsP2 also suppressed the activity of replicases of heterologous alphaviruses. Thus, the presence of nsP2 inhibits formation and activity of alphavirus RNA replicase in protease activity-dependent and -independent manners. This knowledge improves our understanding about mechanisms of superinfection exclusion for alphaviruses and may aid the development of anti-alphavirus approaches.

## 1. Introduction

Alphaviruses are enveloped, positive-strand RNA viruses belonging to family *Togaviridae* [1]. Most known alphaviruses infect vertebrate hosts and are transmitted between them by arthropod vectors, most commonly by mosquitoes. However, alphaviruses restricted to invertebrates have also been discovered [2]. Arthropod-transmitted alphaviruses are divided between several complexes [1]; in addition, they are often divided into Old World and New World alphaviruses. The latter division reflects different pathology in vertebrate hosts, including humans: New World alphaviruses cause encephalitis, while Old World alphaviruses typically cause fever and arthritis. Currently, chikungunya virus (CHIKV) is medically the most important alphavirus. CHIKV uses *Aedes aegypti* and *Aedes albopictus* as transmission vectors [3,4] and can cause outbreaks anywhere these mosquitoes are present. In recent decades, it has caused explosive outbreaks in Africa, Asia, the Indian Ocean region, and the Americas; in addition, there have been small outbreaks in Southern Europe [5,6]. 

The RNA genome of alphaviruses is approximately 12 kb in length, has a cap structure at the 5′end, a polyA tail at the 3′end and contains two open reading frames (ORFs). The first ORF encodes nonstructural (ns) polyproteins P123 and P1234 that are precursors of four ns proteins (nsP1-4), the virus-encoded subunits of the RNA replicase complex (RC) [7]. Mature nsPs are formed by cleavage of ns polyproteins at three cleavage sites designated as the 1/2 (site between nsP1 and nsP2), 2/3 and 3/4 sites; the protease activity resides in the C-terminal part of nsP2 [8,9,10]. The processing of ns polyproteins is well regulated; both the order and timing of cleavages are important for active RNA replicase formation [11,12,13]. The cascade starts with cleavage of the 3/4 site, is followed by cleavage of the 1/2 site that occurs in cis and is completed by cleavage of the 2/3 site that occurs in trans [14,15]. The products of these cleavage steps correspond to different forms of the alphavirus RC. P123 + nsP4 functions as early replicase and is required for negative-strand RNA synthesis. nsP1 + P23 + nsP4 is a short-lived intermediate replicase [16]. During these stages the physical structures of the RC, membrane-bound structures called spherules, are formed [17]. In the final step, P23 is cleaved and mature RCs, containing all four nsPs, are formed. Mature RCs use negative-strand RNA as a template and synthesize positive-strand genomic RNAs and shorter subgenomic (SG) RNAs [16]; the latter are used as mRNAs for translation of structural polyproteins encoded in the second ORF [18]. Interestingly, this is the only ns polyprotein processing pathway known to result in formation of functional RCs: if the P1234 is first cleaved at the 2/3 site, the cleavage products (P12 and P34) fail to form RC and serve as a source of free (not included into functional RCs) nsPs that have multiple functions in infected cells [19]. This change of processing pathway, triggered by accumulation of nsP2 protein in infected cells, is used by alphaviruses to shut down negative-strand RNA synthesis and new RC formation [15,20].

Alphavirus nsP2 is a multifunctional protein. Its N-terminal region has a superfamily 1 RNA helicase fold [21], possesses NTPase and RNA triphosphatase activities [22,23] and, together with the C-terminal region, also has RNA helicase activity [24,25]. The C-terminal region of nsP2 has a papain-like protease fold [26] with an active site containing a conserved catalytic dyad with Cys and His residues. The N- and C-terminal regions of nsP2 are connected by a flexible linker and function in a coordinated manner [25,27]. In addition to enzymatic functions essential for viral genome replication, nsP2 also has functions related to shutoff of host cell transcription and antiviral responses. In vertebrate cells, nsP2 of Old World alphaviruses translocates into the nucleus [28], where it causes degradation of the RPB1 subunit of RNA polymerase II and shutdown of cellular mRNA synthesis [29]. Similarly, nsP2 has been identified as a factor counteracting interferon induction and signaling [30,31]. Several studies have revealed that mutations associated with non-cytotoxic phenotype of alphaviruses or alphavirus-derived self-replicating RNAs (replicons) map to the C-terminal part of nsP2. While many of these mutations also compromise the efficiency of viral RNA replication [32,33,34], mutations blocking the ability of nsP2 of CHIKV or Sindbis virus (SINV) to cause cellular transcription shutoff without affecting viral replication rates have recently been described [35,36]. 

Superinfection exclusion (SIE) is a phenomenon whereby an infected cell becomes resistant to infection by the same or another virus. SIE is common for viruses and was first described for an alphavirus nearly 50 years ago [37]. However, despite half a century of studies, its mechanism(s) are not fully understood, possibly because of differences between different alphaviruses, different (vertebrate and invertebrate) host cells and methodologies used. It has been established that SIE occurs early in alphavirus infection [37]. Cells infected by SINV or CHIKV (first virus) become resistant to subsequent infection (“superinfection”) by SINV, CHIKV or Semliki Forest virus (SFV). Such a phenomenon has been observed with several alphaviruses, including the insect-specific virus Eilat virus (EILV), confirming that one alphavirus can generally block replication of another one [38,39,40]. In superinfected cells, the expression of ns proteins of the incoming alphavirus is allowed [41], indicating that the block occurs at a subsequent, RNA replicase formation/RNA replication stage. The effect has been attributed to the trans-cleavage of ns polyprotein of superinfecting virus by nsP2 of the first virus [39], a hypothesis that is supported by findings that alphaviruses or replicons harboring mutations in nsP2 are often unable to block superinfection by wild-type (wt) virus [42,43] and that nsP2 protease of one alphavirus can cleave the ns polyprotein of another alphavirus at the 2/3 site [14]. However, this may not be the only or universal mechanism behind SIE. Using single-cell technologies, it has been recently found that SIE in CHIKV-infected mammalian cells was not mediated by the action of any single CHIKV ns protein [44]. Using quantitative live-cell and single-molecule imaging, it was demonstrated that SIE represents an indirect phenotypic consequence of a bidirectional competition between the first and superinfecting alphaviruses, coupled with the rapid onset of viral replication and a limited total cellular carrying capacity [45]. 

In this study, we addressed the role of alphavirus nsP2 and its protease activity in interfering with the activity of alphavirus RNA replicase in *Aedes albopictus* mosquito cells. Mosquito cells were chosen in order to include the insect-specific EILV and to avoid nsP2-mediated cytotoxicity common for mammalian cells; furthermore, follow-up in vivo studies using transgenic organisms are much more feasible for mosquitoes than for vertebrates. A recently developed trans-replication system [46] was used to provide viral RNA replicase and measure its activities; this system also allows the use of mutations in ns polyprotein in the absence of reversions/second-site compensatory changes. It was observed that co-expression of nsP2, lacking protease activity due to replacement of a catalytic Cys residue with Ala (nsP2^CA^) reduced the activity of trans-replicase of homologous and heterologous alphaviruses. This indicates the existence of protease activity-independent inhibition, which may represent a consequence of co-expressed free nsP2^CA^ interfering with RC formation and functioning. In addition, such an inhibition can also be mediated by changes induced by excess free nsP2 in the transfected cells. However, the ability of wt nsP2 to inhibit activities of trans-replicases of homologous and heterologous alphaviruses was always higher. The same was observed in experiments in which CHIKV RNA replicons packaged into virus replicon particles (VRPs) were used to infect nsP2 expressing cells. The ability of nsP2 to block activity of trans-replicase was minimally, if at all, affected by mutations increasing protease activity of nsP2, reducing cytotoxicity of nsP2 or altering its subcellular localization. Importantly, the ability of nsP2 to cause reduction of RNA replication in a protease activity-dependent manner depended on its ability to process the precursor of the replicase. Thus, a negative impact resulting from co-expression of nsP2 was reduced by mutation-blocking cleavage of the 1/2 site in the ns polyprotein and essentially eliminated by mutation-blocking cleavage of the 2/3 site. These data unequivocally demonstrate that, in this assay system, protease activity of free nsP2 contributes to blocking the formation of functional RCs and infection of cells by packaged CHIKV replicons. It remains unclear whether these effects are identical to those occurring in superinfected cells in which the amounts of free nsP2 and ns polyproteins are different from those used in our assays. However, these data open interesting possibilities for engineering of artificial broad-spectrum alphavirus resistance in mosquitoes. 

## 2. Materials and Methods

### 2.1. Cell Lines

*Aedes albopictus*-derived C6/36 cells were maintained in Leibovitz’s L-15 medium (PAN Biotech, Aidenbach, Germany) supplemented with 10% heat-inactivated fetal bovine serum (FBS) and 1% tryptose phosphate broth (TPB) at 28 °C without additional CO_2_. BHK-21 cells (ATCC, CCL-10) were cultured in Glasgow’s minimal essential medium (GMEM; Thermo Fisher Scientific, Waltham, MA, USA) supplemented with 10% FBS and 2% TPB at 37 °C in a humidified incubator with 5% CO_2_. All growth media were supplemented with 100 U/mL of penicillin and 0.1 mg/mL of streptomycin (Thermo Fisher Scientific, Waltham, MA, USA). 

### 2.2. Construction of nsP2 Expression Plasmids

Sequences encoding for 10 C-terminal amino acid residues of nsP1 and full-length nsP2 of CHIKV (isolate LR2006OPY1) or nsP2 of SINV (isolate Toto1101) harboring Asn614 to Asp mutation (Table 1) were optimized according to codon usage of *Aedes aegypti*, and the cryptic splicing sites present in these sequences were also removed. Corresponding synthetic DNAs were obtained from Twist Bioscience (San Francisco, CA, USA) and cloned between *Aedes aegypti* polyubiquitin promoter and transcription terminator of hsp70 gene from *Drosophila melanogaster* in the plasmid pB-HR5/IE1.DsR that also contains cassette for expression of red fluorescent protein DsRed. Obtained plasmids were designated as pPubi-CHIKV-nsP2 and pPubi-SINV-nsP2^ND^. Asn614 to Asp mutation in SINV nsP2 was removed using site-directed mutagenesis, and the resulting plasmid was designated as pPubi-SINV-nsP2. Other mutations affecting nsP2 protease activity, cytotoxicity or sequence corresponding to nuclear localization signal (NLS) in nsP2 of SFV (Table 1) were introduced into pPubi-CHIKV-nsP2 and pPubi-SINV-nsP2 using PCR based site-directed mutagenesis and subcloning. The resulting clones were designated as pPubi-CHIKV-nsP2^CA^, pPubi-CHIKV-nsP2^EV^, pPubi-CHIKV-nsP2^YA+EV^, pPubi-CHIKV-nsP2^KR/DD^, pPubi-CHIKV-nsP2^ALT/ERR^, pPubi-SINV-nsP2^CA^, pPubi-SINV-nsP2^KR/DD^, pPubi-SINV-nsP2^PQ^ and pPubi-SINV-nsP2^ND+PQ^. Sequences of all plasmids were confirmed using Sanger sequencing and are available from authors upon request.

### 2.3. Analysis of nsP2 Expression

C6/36 cells grown in 6-well plates were transfected with 800 ng of pPubi-CHIKV-nsP2 or pPubi-SINV-nsP2 using Lipofectamine LTX and the Plus Reagent kit (Thermo Fisher Scientific, Waltham, MA, USA). Cells were harvested at 12, 18, 24, 36 and 48 h post transfection (hpt), lysed with 1× Laemmli buffer (50 mM Tris-HCl [pH 6.8], 2% SDS, 10% glycerol, 100 mM dithiothreitol, and 0.1% bromophenol blue) and boiled for 10 min. Proteins were separated by SDS-PAGE in 10% gels, transferred to polyvinylidene difluoride (PVDF) membrane followed by staining with primary anti-CHIKV nsP2 (in-house, raised against recombinant protein corresponding to residues 1–470 of nsP2 of CHIKV) or anti-SINV nsP2 (in-house, raised against full-length recombinant nsP2 of SINV) antibodies and antibody against β-actin (sc-47778; Santa Cruz Biotechnology, Dallas, TX, USA). After washing, the membrane was incubated with the appropriate secondary antibodies conjugated to fluorescent infrared dyes (LI-COR. Lincoln, NE, USA), and proteins were detected using the LI-COR Odyssey Fc imaging system. In another experiment, C6/36 cells grown in 6-well plates were transfected with 800 ng pPubi-CHIKV-nsP2, pPubi-CHIKV-nsP2^CA^, pPubi-CHIKV-nsP2^EV^, pPubi-CHIKV-nsP2^YA+EV^, pPubi-CHIKV-nsP2^KR/DD^, pPubi-CHIKV-nsP2^ALT/ERR^, pPubi-SINV-nsP2, pPubi-SINV-nsP2^CA^, pPubi-SINV-nsP2^ND^, pPubi-SINV-nsP2^KR/DD^, pPubi-SINV-nsP2^PQ^ or pPubi-SINV-nsP2^ND+PQ^ using Lipofectamine LTX and the Plus Reagent kit. Cells were harvested at 48 hpt, lysed and analyzed as described above. 

### 2.4. Trans-Replicase Assay

The expression constructs of CHIKV ns polyprotein (Ubi-P1234-CHIKV), its variants harboring mutations blocking the cleavage at 1/2 site (Ubi-P1^GV^234-CHIKV) or at 2/3 site (pUbi-P12^GV^34-CHIKV) and plasmid expressing ns polyprotein harboring inactivating mutation in the RNA polymerase active site of nsP4 (Ubi-P1234^GAA^-CHIKV) have been previously described [47]. Plasmid for the expression of replication-competent template RNA of CHIKV harboring firefly luciferase (Fluc) and Gaussia luciferase (Gluc) markers using *Aedes albopictus* RNA polymerase I-based transcription (Alb-FG-CHIKV), has been described in [48], and trans-replicase system plasmids of SINV, SFV, EILV, Ross River virus (RRV), Mayaro virus (MAYV), and Venezuelan equine encephalitis virus (VEEV) in [46]. Plasmids expressing ns polyprotein, its polymerase negative form and template RNA of eastern equine encephalitis virus (EEEV) have a similar design. 

Trans-replication assays were performed as follows: C6/36 cells grown in 96-well plates (35,000 cells/well) were transfected with a mixture containing 440 ng of the ns poly-protein-encoding plasmid (Ubi-P1234-CHIKV, Ubi-P1234^GAA^-CHIKV and so on), 440 ng of plasmid expressing corresponding template (Alb-FG-CHIKV and so on) and 440 ng plasmid encoding nsP2 of CHIKV or SINV or mutant version thereof. In experiments aiming for the analysis of an effect of co-expression of SINV and CHIKV nsP2 on the RNA replicase from heterologous alphavirus, the matching pairs of ns polyprotein and template RNA expression plasmids were used, and the amount of protease expression plasmids was increased to 1600 ng. In all control experiments, the protease-expressing plasmid was substituted by the same amount of irrelevant (“dummy”) plasmid DNA encoding only DsRed. Transfections were performed using Lipofectamine LTX and Plus Reagent kit according to the manufacturer’s instructions. Cells were harvested at 48 hpt, washed with phosphate-buffered saline (PBS) and lysed using 1× Passive lysis buffer (Promega, Madison, WI, USA). Fluc and Gluc activities were measured using a Dual-Luciferase-Reporter assay kit (Promega), white OptiPlates (PerkinElmer, Waltham, MA, USA) and a Glomax SIS luminometer (Promega). All transfections were performed in three biological replicates. Raw data is presented in Table S1.

### 2.5. Packaging of CHIKV Replicons into Virus Replicon Particles (VRPs)

A two-helper system for CHIKV replicons has been described previously [49]. Plasmid pSP6-CHIKVRepl-ZsGreen was constructed by cloning sequence encoding for ZsGreen marker under the control of SG promoter in CHIKV replicon vector. pSP6-CHIKVRepl-ZsGreen and helper plasmids were linearized with NotI. DNA was purified and transcribed in vitro using an mMESSAGE mMACHINE SP6 transcription kit (Ambion, Austin, TX, USA). Then, 8 × 10^6^ BHK-21 cells were transfected via electroporation (850 V, 25 µF, two pulses in a cuvette with a 4-mm electrode gap) with 1 µg of RNA transcripts corresponding to the replicon and each helper RNA. The transfected cells were seeded in a 60-mm plate; after incubation of the plates at 37 °C for 48 h, supernatants containing VRPs were harvested and clarified by centrifugation at 1000× *g* for 10 min. For determination of the titer, different dilutions of VRP stock were made in L-15 medium and used for infection of C6/36 cells. Cells were incubated for 16 h at 28 °C, harvested and percentage of cells expressing ZsGreen was determined using flow cytometry as described below.

### 2.6. Infection of Transfected Cells with VRPs and Flow Cytometry Assay

C6/36 cells were grown in 12-well plates at 350,000 cells/well and transfected with 3 µg pPubi-CHIKV-nsP2, pPubi-CHIKV-nsP2^CA^, pPubi-SINV-nsP2, pPubi-SINV-nsP2^CA^ or pB-HR5/IE1.DsR (empty expression vector) using Lipofectamine LTX and the Plus Reagent kit (Thermo Fisher Scientific). At 48 hpt cells were infected with CHIKV VRPs at multiplicity of infection of approximately 0.4 infectious VRP/cell and incubated for 16 h at 28 °C. Afterward, medium was removed, cells were fixed by incubation at room temperature in 10% formalin for 30 min. Fixed cells were washed 3 times with PBS and suspended in 1 mL of PBS. Cell analysis was performed using Attune NxT Acoustic Focusing Cytometer (Thermo Fisher Scientific). For detecting ZsGreen fluorescent signal, 488 nm laser with 493–490 filter was used, while detection of DsRed was performed using a 561-nm laser with a 612–627 filter. A scatter plot was used to determine the percentage of viable cells in the prepared samples. For each sample, 30,000 events were recorded. The experiment was performed in triplicates and repeated two times. Raw data is presented in File S1.

### 2.7. Statistical Analysis

Statistical analysis was performed with GraphPad Prism 8.2.0 software. Data were analyzed using one-way ANOVA test. *p*-values < 0.05 were considered statistically significant.

**Table 1:** Mutations introduced into nsP2 of CHIKV and SINV.

Mutation	Position(s)	Original Residue(s)	Mutated Residue(s)	Effect (Reference)
CHIKV-nsP2^CA^	478	Cys	Ala	Inactivates protease [8]
CHIKV-nsP2^EV^	515	Glu	Val	Activates ns polyprotein processing [47]
CHIKV-nsP2^YA+EV^	161 and 515	Tyr (161), Glu (515)	Ala (161), Val (515)	Blocks RNA replication [21] and activates ns polyprotein processing [47]
CHIKV-nsP2^KR/DD^	649–650	Lys-Arg	Asp-Asp	Changes sequence corresponding to NLS in SFV nsP2 [50]
CHIKV-nsP2^ATL/ERR^	674–676	Ala-Thr-Leu	Glu-Arg-Arg	Blocks RPBI degradation [35]
SINV-nsP2^CA^	481	Cys	Ala	Inactivates protease [51]
SINV-nsP2^ND^	614	Asn	Asp	Hyper-activates nsP2 protease [51]
SINV-nsP2^KR/DD^	658–659	Lys-Arg	Asp-Asp	Changes sequence corresponding to NLS in SFV nsP2 [50]
SINV-nsP2^PQ^	683	Pro	Gln	Blocks RPBI degradation [36]
SINV-nsP2^ND+PQ^	614 + 683	Asn (614), Pro (683)	Asp (614), Gln (683)	Hyper-activates nsp2 protease [51] and blocks RPBI degradation [36]

## 3. Results

### 3.1. Trans-Replicases of Alphaviruses and nsP2 Expression Plasmids

**Figure 1:**
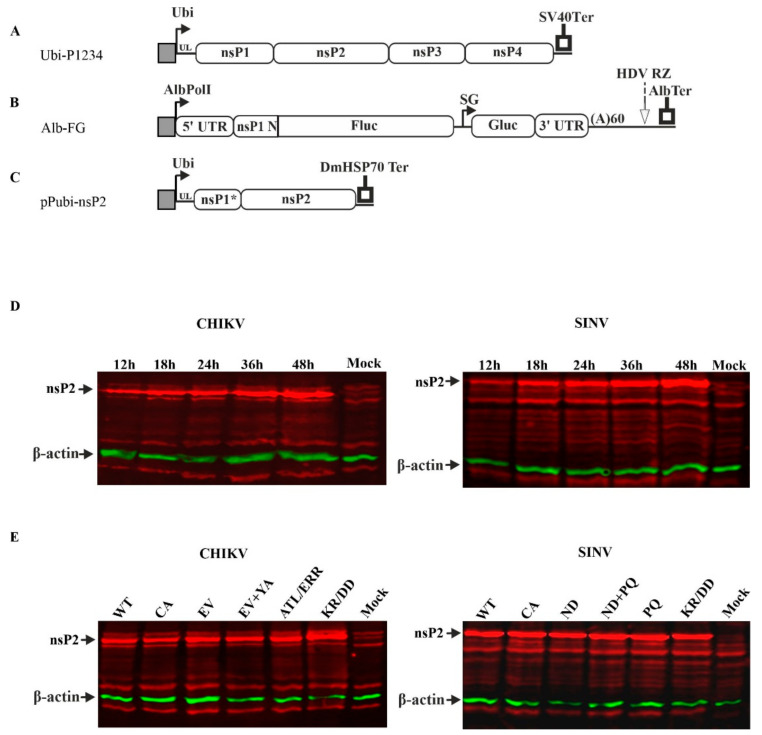
Schematic overview of used plasmids and verification of nsP2 expression in transfected C6/36 cells. (**A**). Trans-replicase plasmid for expression of alphavirus ns-polyprotein. Ubi—full-length *Aedes aegypti* polyubiquitin promoter; UL—transcribed leader of polyubiquitin gene containing naturally occurring intron; SV40Ter—SV40 late polyadenylation region. (**B**) Constructs expressing template RNAs for trans-replicases. AlbPolI—truncated (−250 to −1) promoter for *Aedes albopictus* RNA polymerase I; AlbTer—tentative terminator for *Aedes albopictus* RNA polymerase I. The 5′ and 3′ UTRs and SG promoter are from CHIKV, SINV, SFV, RRV, MAYV, EILV, VEEV or EEEV; nsP1N—region encoding for the N-terminal region of nsP1; HDV RZ—antisense strand ribozyme of hepatitis delta virus. (**C**). Constructs expressing nsP2 of CHIKV or SINV. nsP1*—region encoding for 10 C-terminal amino acid residues of nsP1; DmHSP70Ter—transcription terminator of *Drosophila melanogaster* hsp70 gene. (**A**–**C**). The vector backbones are not shown; drawings are not in scale. (**D**). C6/36 cells were transfected with pPubi-CHIKV-nsP2 (left) or pPubi-SINV-nsP2 (right). Cells were harvested at 12, 18, 24, 36 or 48 hpt and lysed in 1× Laemmli buffer. Proteins were separated using SDS-PAGE in 10% gels and transferred to PVDF membranes. nsP2 proteins were detected using anti-CHIKV and anti-SINV nsP2 antibodies, and β-actin was detected as the loading control. (**E**) C6/36 cells were transfected with (left panel) pPubi-CHIKV-nsP2 (WT), pPubi-CHIKV-nsP2^CA^, pPubi-CHIKV-nsP2^EV^, pPubi-CHIKV-nsP2^YA+EV^, pPubi-CHIKV-nsP2^ALT/ERR^, pPubi-CHIKV-nsP2^KR/DD^; (right panel) pPubi-SINV-nsP2 (WT), pPubi-SINV-nsP2^CA^, pPubi-SINV-nsP2^ND^, pPubi-SINV-nsP2^ND+PQ^, pPubi-SINV-nsP2^PQ^ or pPubi-SINV-nsP2^KR/DD^. Cells were harvested at 48 hpt and analyzed as described for (**D**).

In order to analyze the impact of nsP2 co-expression on the formation and function of RNA replicase using infectious viruses and standard cell culture techniques, one needs to ensure the nsP2 expression in the majority of cells. Unfortunately, transfection of mosquito cells with expression plasmids using available transfection reagents is relatively inefficient [46] and efficiencies close to 100% are not achievable. Our attempts to circumvent this issue by construction of stable mosquito cell lines for expression nsP2 of CHIKV or SINV were not successful: in the cell lines obtained, no expression of nsP2 was observed. To overcome these issues, advantage was taken of the recent development of alphavirus trans-replicase systems that are highly active in *Aedes albopictus* cells [46]. In this system, ns polyprotein and replication-competent RNA template are generated using separate expression plasmids (Figure 1A,B). When co-transfected into mosquito cells, these plasmids express viral components that assemble into functional RCs similar to those seen in infected cells [52,53]. This makes this trans-replicase system a highly relevant tool to study alphavirus RNA replication as well as mutations inhibiting RC-formation/functioning [21,54]. The system also benefits from a lack of reversions/pseudoreversions and from simplicity of measurement of efficacy of RNA synthesis. For this purpose, the first ORF in the replicating RNA, with the exception of a few hundreds of 5′ residues forming structures essential for RNA replication, is replaced with sequence encoding for Fluc, and the entire second ORF is replaced with sequence encoding Gluc. For simplicity, and following [46], hereafter, synthesis of the RNA serving as template for Fluc expression is termed as “replication” and synthesis of RNA serving as template for Gluc expression from the SG promoter as “transcription”. The efficiency of replication and transcription were estimated by fold changes (“boost”) of corresponding reporter expression i.e., reporter activity in cells expressing native P1234 of alphavirus relative to those expressing its polymerase-negative P1234^GAA^ variant [46]. Such systems were available for several viruses belonging to SFV complex: CHIKV, SFV, RRV and MAYV; for SINV, belonging to western equine encephalitis complex, for VEEV belonging to Venezuelan equine encephalitis complex, for EEEV belonging to eastern equine encephalitis complex and for insect-specific EILV [46].

To initiate RNA replication, a cell must be co-transfected with ns polyprotein and template RNA expression plasmids. Importantly, we have previously observed that splitting the ns polyprotein expression cassette between two plasmids does not reduce trans-replicase activity, indicating high efficiency of co-transfection of three plasmids [55]. Therefore, the trans-replicase represents a suitable system for analysis of an impact of co-expressed nsP2 on formation/functioning of alphavirus RNA replicase. Importantly, activity of alphavirus nsP2 critically depends on its N-terminal residue that is Gly for nsP2 of CHIKV and Ala for nsP2 of SINV. Any modification of this residue or extension/truncation of the N-terminus of nsP2 has severe impact on the ability of nsP2 to cleave the 2/3 site in ns polyproteins [11,14] and can compromise other activities of the protein [25]. For these reasons, use of artificial start codon to express nsP2 inevitably results in expression of flawed enzyme and consequently may lead to experimental artifacts. To avoid such problems, we extended the 5′ region of the expression construct to also encode 10 C-terminal residues of nsP1 (Figure 1C); these residues are subsequently removed by protease activity of nsP2. This approach results in proteins with authentic N-termini generated via proteolysis of the 1/2 site, as for alphavirus-encoded nsP2 in infected cells.

The ability of the newly constructed plasmids to express nsP2 in transfected C6/36 cells was validated using immunoblotting. Transfected cells were harvested, and expressed proteins were analyzed at different time points. It was observed that expression of both nsP2 of CHIKV and SINV was detectable as early as 12 hpt and that the concentration of expressed protein increased further over 48 h (Figure 1D; File S2). As 48 hpt is also the preferred time point for measurement of trans-replicase activity in *Aedes albopictus* cells [46], it was used in all subsequent experiments. 

Over years of study, numerous mutations in nsP2 of CHIKV and SINV that affect its protease activity, subcellular localization or ability to shutoff transcription in vertebrate cells have been described (Table 1 and references within). These mutations can potentially affect the ability of nsP2 to interfere with alphavirus RNA replicase formation/activity, and therefore, expression plasmids for several of such mutant proteins were also constructed. The list included nsP2 with mutation of the catalytic Cys residue of protease active site to Ala, which completely inactivates its protease activity [8] and therefore allows discrimination between effects caused by the presence of nsP2 and those caused by its protease activity. The expression of all mutant nsP2 proteins was verified using immunoblotting. The expression levels of mutant proteins were found to be similar to those of wt counterparts (Figure 1E; File S2), indicating that introduced substitutions had no major effect on the expression level or stability of mutant proteins.

### 3.2. Co-Expression of nsP2 of CHIKV and SINV Inhibits Activities of Corresponding Trans-Replicases

Formation of functional RNA replicase of alphaviruses requires perfectly timed processing of ns polyproteins by the protease activity of nsP2 precursors and mature nsP2 [11]. The timeliness of processing depends on catalytic activity of nsP2 protease, accessibility of cleavage sites and, for trans-cleavages, enzyme-to-substrate ratio. Elevated levels of mature nsP2 or increased catalytic activity of nsP2 protease should therefore accelerate processing of ns polyproteins at the 2/3 site, which is cleavable in trans, and consequent inhibition of RNA replicase formation. In addition, excess free nsP2 in cells may interfere with activity of viral RNA replicase in a protease activity-independent manner by altering the stoichiometry of subunits of RNA replicase or by binding host factor(s) required for RNA replicase activities. Furthermore, free nsP2 may also affect RNA replication indirectly by affecting host cell responses and/or altering its viability. 

**Figure 2:**
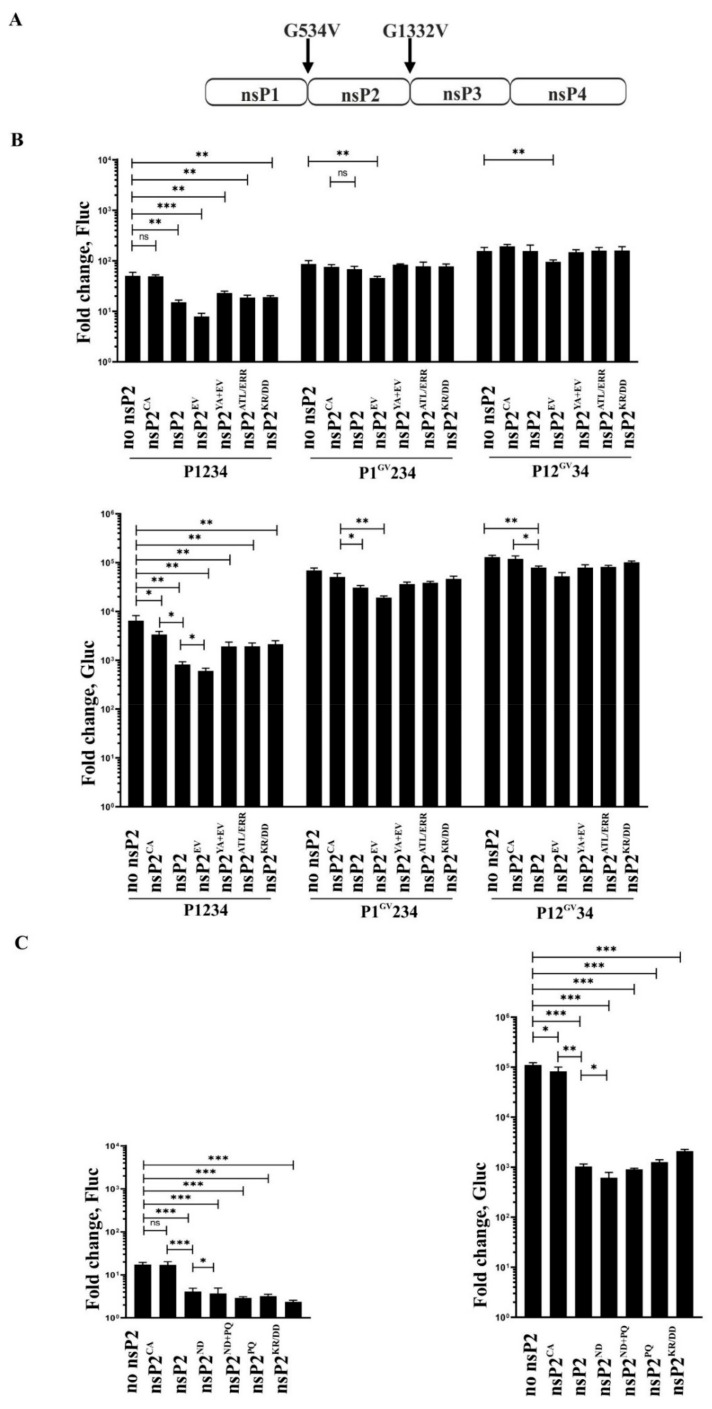
Co-expression of CHIKV and SINV nsP2 inhibits activity of trans–replicase of homologous virus. (**A**). Mutations introduced to ns-polyprotein of CHIKV. Mutation G534V leads to expression of P1^GV^234 polyprotein with uncleavable 1/2 processing site, while mutation G1332V leads to expression of P12^GV^34 polyprotein with uncleavable 2/3 processing site. (**B**). C6/36 cells grown at 96-well plate were co-transfected with Alb-FG-CHIKV, wt or mutant ns-polyprotein expression plasmid (Ubi-P1234-CHIKV, Ubi-P1^GV^234-CHIKV, Ubi-P12^GV^34-CHIKV or Ubi-P1234^GAA^-CHIKV) and pPubi-CHIKV-nsP2^CA^, pPubi-CHIKV-nsP2, pPubi-CHIKV-nsP2^EV^, pPubi-CHIKV-nsP2^YA+EV^, pPubi-CHIKV-nsP2^ALT/ERR^, pPubi-CHIKV-nsP2^KR/DD^ or with dummy plasmid (no-nsP2 control). Cells were incubated at 28 °C and lysed 48 hpt. Data represent the luciferase activity (Fluc and Gluc) from Ubi-P1234-CHIKV (left), Ubi-P1^GV^234-CHIKV (middle) and Ubi-P12^GV^34-CHIKV (right) transfected cells normalized to the Ubi-P1234^GAA^ control cells. Value obtained for P1234^GAA^ control was taken as 1. (**C**). C6/36 cells grown at 96-well plate were co-transfected with Alb-FG-SINV, Ubi-P1234-SINV or Ubi-P1234^GAA^-SINV and pPubi-SINV-nsP2^CA^, pPubi-SINV-nsP2, pPubi-SINV-nsP2^ND^, pPubi-SINV-nsP2^ND+PQ^, pPubi-SINV-nsP2^PQ^, pPubi-SINV-nsP2^KR/DD^ or with dummy plasmid (no-nsP2 control). The experiment was performed and data analyzed as described for panel B. (**B**,**C**). Means ± SD from three biological replicates are shown. ns, not significant, * *p* < 0.05, ** *p* < 0.01, *** *p* < 0.001 (one-way ANOVA test).

In order to analyze the impact of alphavirus nsP2 on the activity of CHIKV RNA replicase, we transfected C6/36 cells with an expression plasmid for wt nsP2 or its protease inactive form (nsP2^CA^) and plasmids for CHIKV trans-replicase. It was observed that if these plasmids were used in equal quantities, the expression of nsP2^CA^ had no impact on RNA replication and caused only minor (approximately 1.8-fold) reduction of transcription. In contrast, expression of wt nsP2 resulted in approximately 4-fold reduction of replication and 7-fold reduction of transcription (Figure 2B), indicating that protease activity of co-expressed nsP2 was crucial for inhibition of activities of CHIKV trans-replicase (Figure 2B).

The substitution of Glu515 residue to Val is associated with increased protease activity of nsP2 of CHIKV and SFV [11,47,56]. Introduction of this mutation into the nsP2 expression construct increased the negative impact of co-expressed protein on CHIKV trans-replicase activities: in the presence of nsP2^EV^, approximately 6-fold reduction of replication and 13-fold reduction of transcription were observed. To find out whether or not this effect was indeed associated with increased catalytic activity of nsP2 protease, a Tyr161 to Ala substitution was introduced into nsP2^EV^. This mutation in the helicase region of nsP2 affects its interaction with RNA. It completely abolishes the ability of CHIKV replicase, harboring such substitution, to perform RNA synthesis [21], but likely does not affect protease activity. It was found that the negative effect of co-expression nsP2^AY+EV^ on the activities of CHIKV trans-replicase was similar to that of co-expression of wt nsP2 (Figure 2B). This data suggests that the more prominent negative impact of nsP2^EV^ on CHIKV RNA replication was not caused by the increased protease activity of co-expressed protein; however, it cannot be excluded that two mutations present in nsP2^AY+EV^ caused independent effects of opposite polarity that cancelled each other. In contrast to the Glu515 to Val, introduction of mutations causing reduction of cytotoxic properties of nsP2 [35] or mutation-eliminating putative NLS in nsP2 did not alter ability of co-expressed nsP2 to reduce activity of CHIKV trans-replicase (Figure 2B).

The role of protease activity of co-expressed nsP2 for inhibition of CHIKV trans-replicase activities was confirmed using mutations introduced into the ns polyprotein expression construct. The Gly534 to Val substitution (resulting in P1^GV^234 polyprotein) blocks cleavage of ns polyprotein at the 1/2 site, while Gly1332 to Val substitution (resulting in P12^GV^34 polyprotein) blocks the cleavage of the 2/3 site (Figure 2A). Consistent with a previous report [47], both of these mutations increased activities of CHIKV trans-replicase in C6/36 cells, the impact of Gly1332 to Val substitution being more prominent (Figure 2B). Both mutations also reduced the sensitivity of CHIKV trans-replicase to the negative impact caused by co-expressed nsP2 or its mutants. With the exception of nsP2^EV^, the impact of co-expression of any of these proteins on the replicase activity of CHIKV trans-replicase harboring Gly534 to Val substitution was minor; no negative effect on the replicase activity of trans-replicase harboring Gly1332 to Val substitution was observed. The nsP2^EV^, however, caused significant reduction of activities of both mutant trans-replicases; this is consistent with the protease activity-independent dominant negative effect of this mutant protein. The impact of nsP2 co-expression on transcription activities of CHIKV trans-replicase was also reduced by the mutations in the 1/2 or 2/3 cleavage sites. The negative impact of co-expression of most of mutant nsP2 proteins was minimal and similar to that resulting from nsP2^CA^ co-expression, indicating that the observed impact was mostly, if not exclusively, protease-activity independent. However, an impact of wt nsP2 co-expression on transcriptase activities of both mutant trans-replicases slightly but significantly exceeded that of nsP2^CA^ (Figure 2B). It is plausible that this effect was not due to inhibition of RNA replicase formation. nsP2 of alphaviruses has a specific role in SG RNA synthesis [57], and it is possible that excess of co-expressed wt nsP2 specifically interfered with this activity. Taken together, it was found that introduction of mutation into the 2/3 site diminished or eliminated the negative impact of protease activity of co-expressed nsP2 on CHIKV trans-replicase. The block of 1/2 cleavage resulted in a similar, albeit slightly smaller effect, most likely because the cleavage at the 2/3 site is affected by previous processing of the 1/2 site [15]. Again, these data support an important role of P1234 processing by protease activity of free nsP2 for blocking the formation of functional RCs. However, we cannot exclude the possibility that the strongly elevated RNA replication and SG RNA transcription levels observed in the presence of P1^GV^234 or P12^GV^34 polyproteins (Figure 2B) may also reduce sensitivity of the used assay, thus diminishing the apparent impact from nsP2 co-expression on RNA replication.

To determine whether the observed effects were specific for CHIKV, or alternatively that they are also observed with other alphaviruses, the experiments were additionally performed using SINV trans-replicase and plasmids expressing SINV wt nsP2 and its mutant versions (Table 1). The results obtained for SINV trans-replicase were extremely similar to those for CHIKV trans-replicase. Under the conditions used, co-expression of nsP2^CA^ had no detectable impact on replication and caused only minor decrease of transcription. In contrast, expression of wt nsP2 caused significant, approximately 4-fold reduction of replication and even more prominent 27-fold reduction of transcription, confirming an important role of the protease activity of co-expressed nsP2 in the inhibition of SINV trans-replicase activities (Figure 2C). nsP2^ND^, harboring a mutation shown to hyper-activate protease activity of nsP2 of SINV, caused a slightly more prominent effect: this mutant reduced the replication approximately 5-fold and transcription 42-fold. nsP2 harboring a mutation reducing its cytotoxicity caused a similar, approximately 5-fold, reduction of replication activity but was a slightly less effective inhibitor of transcription (approximately 25-fold reduction was observed). Effects caused by co-expression of nsP2^ND+PQ^ were similar to those caused by co-expression of nsP2^ND^. Finally, a mutation introduced to putative NLS of nsP2 of SINV slightly increased its ability to reduce the replication but decreased its ability to inhibit transcription (Figure 2C).

Taken together, this data clearly demonstrates that co-expression of nsP2 inhibits formation and/or activity of RNA replicase of homologous alphavirus using different mechanisms. Of these, the cleavage of synthesized ns polyproteins between nsP2 and nsP3 regions by protease activity of co-expressed nsP2 has the largest impact.

### 3.3. Expression of nsP2 Inhibits Infection of C6/36 Cells by CHIKV VRPs

Trans-replicases of alphaviruses serve as excellent surrogate models to study RNA replicase activities. However, if they are used to mimic alphavirus superinfection, an important limitation applies: plasmid expressing nsP2 (mimicking the first alphavirus infecting a cell) and plasmids of trans-replication system (mimicking superinfecting virus) are co-transfected, i.e., not delivered in a sequential manner as it is the case for superinfection. However, even with this limitation, our data (Figure 2B,C) strongly suggest that nsP2 and its protease activity are very likely involved in SIE. To provide an experimental system more similar to natural virus infection, advantage was taken of the red fluorescent marker DsRed co-expressed from nsP2-expressing plasmids, allowing identification of transfected (i.e., nsP2-expressing) cells and CHIKVRepl-ZsGreen replicon that could be packaged into VRPs and, in VRP-infected cells, expresses green fluorescent marker ZsGreen. Together, these two markers allow identification of cells that are transfected with nsP2 expression construct and successfully infected with VRPs. In addition, CHIKV replicons are single-cycle vectors, do not spread in cell culture and allow accurate measurement of VRP-infected cell numbers. The conditions of the experiment were selected to achieve approximately 15% of cells to be transfected by each used plasmid, as indicated by expression of DsRed; these levels did not damage transfected cells but were sufficient for analysis of transfected cell populations. VRPs were used in an amount that in non-transfected cells resulted in infection of approximately 40% of cells (i.e., the infection with VRPs mimicked CHIKV infection at moderate multiplicity). 

**Figure 3:**
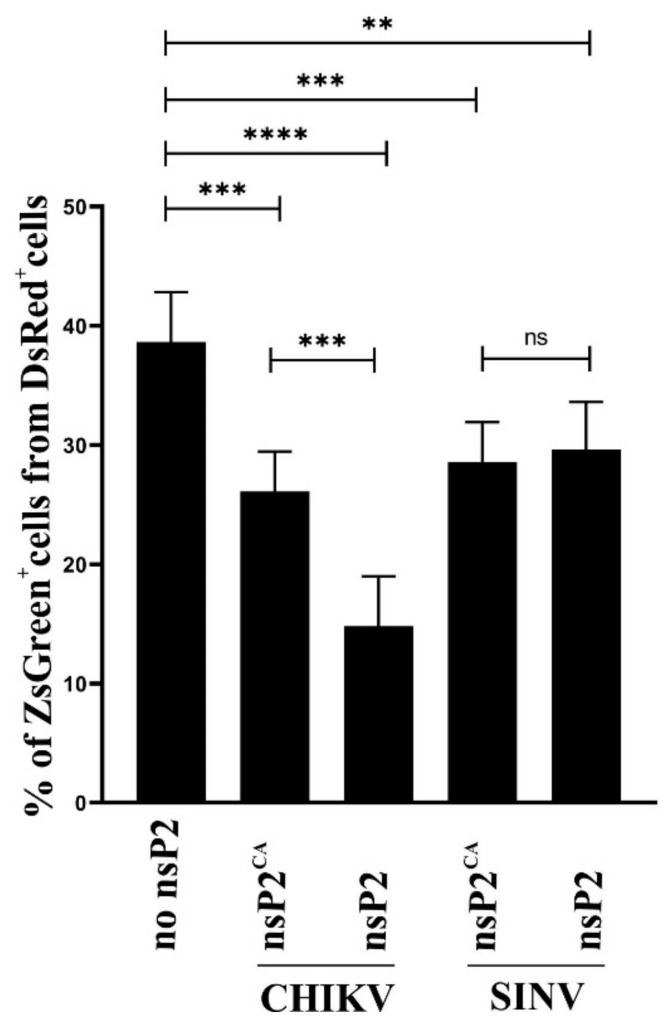
Efficiency of infection of C6/36 cells by CHIKV VRPs is reduced by expression of CHIKV or SINV nsP2 proteins. C6/36 cells grown in 12-well plates were transfected with pB-IE1.dsR (no nsP2), pPubi-CHIKV-nsP2^CA^, pPubi-CHIKV-nsP2, pPubi-SINV-nsP2^CA^ or pPubi-SINV-nsP2. At 48 hpt, cells were infected with VRPs containing CHIKVRepl-ZsGreen replicon at a multiplicity of infection of approximately 0.4. Cells were harvested at 16 h post-infection and fixed and analyzed with an Attune NxT acoustic focusing cytometer. Y-axes: percentage of ZsGreen-positive cells (i.e., harboring replicating CHIKV replicon) from DsRed-positive cells (i.e., cells successfully transfected with nsP2 expression or control plasmid). Means ± SD from two independent experiments performed in triplicate are shown. ns, not significant, ** *p* < 0.01, *** *p* < 0.001, **** *p* < 0.0001 (one-way ANOVA test).

When C6/36 cells were transfected with control plasmid (expressing only DsRed) and infected with VRPs at 48 hpt, expression of ZsGreen was observed in approximately 40% of transfected cells (Figure 3), indicating that transfection and DsRed expression had no negative effect on infection. If the cells were transfected with pPubi-CHIKV-nsP2^CA^ the level of ZsGreen-positive cells dropped to approximately 26% of DsRed-expressing cells (Figure 3). Significantly more prominent reduction was observed when cells were transfected with pPubi-CHIKV-nsP2: the level of ZsGreen-positive cells dropped to approximately 14% of DsRed-expressing cells (Figure 3). In each case, the percentage of VRP-infected DsRed negative cells did not significantly differ between experiments performed using control, pPubi-CHIKV-nsP2^CA^ or pPubi-CHIKV-nsP2 plasmids. These data clearly demonstrate that expression of nsP2 and its protease activity reduced the efficiency of CHIKV infection, consistent with conclusions from the trans-replicase assay above. 

The same experimental system was also used to reveal whether expression of SINV nsP2 has an impact on infection with CHIKV VRPs. It was observed that the transfection of cells with pPubi-SINV-nsP2^CA^ resulted in similar reduction of VRP infection as did transfection with pPubi-CHIKV-nsP2^CA^. However, no further reduction was observed if the cells were transfected with pPubi-SINV-nsP2 (Figure 3). These data indicate that, while the presence of SINV nsP2 did have a negative impact on CHIKV infection in C6/36 cells, this effect was not, at least under these experimental conditions, enhanced by protease activity of SINV nsP2.

### 3.4. Transcription Activities of Trans-Replicases of Heterologous Alphaviruses Are Suppressed by Co-Expression of nsP2 of CHIKV or SINV

Results from experiments performed with CHIKV VRPs were consistent with those obtained using the trans-replicase system (Figure 2 and Figure 3), confirming that trans-replicases can be used as models for analysis of an impact of nsP2 co-expression on RNA synthesis of homologous and heterologous alphaviruses. Furthermore, it was observed that CHIKV infection was also inhibited by expression of SINV nsP2; this observation is consistent with previous reports that SINV infection does interfere with infection of other alphaviruses [38,39]. We therefore analyzed the impact of co-expressed nsP2^CA^ or wt nsP2 of CHIKV or SINV on the activities of trans-replicases of eight different alphaviruses: CHIKV, SINV, SFV, RRV, MAYV, EILV, VEEV and EEEV. When these experiments were performed using the same conditions as described for matching combinations of nsP2 and trans-replicase (i.e., cells were co-transfected with equal amounts of protease and trans-replicase expression plasmids), the effects of nsP2 co-expression on activities of heterologous trans-replicases were small, and consistent results could not be obtained. Therefore, we increased the amount of protease-expressing plasmids 3.6-fold; this did not result in visually detectable damage of transfected cells. As we have previously observed that in C6/36 cells the expression of Fluc marker does not serve as reliable marker of replication for several alphaviruses that were included in this study [46], only an impact of nsP2 co-expression on transcription (Gluc expression) was analyzed. 

**Figure 4:**
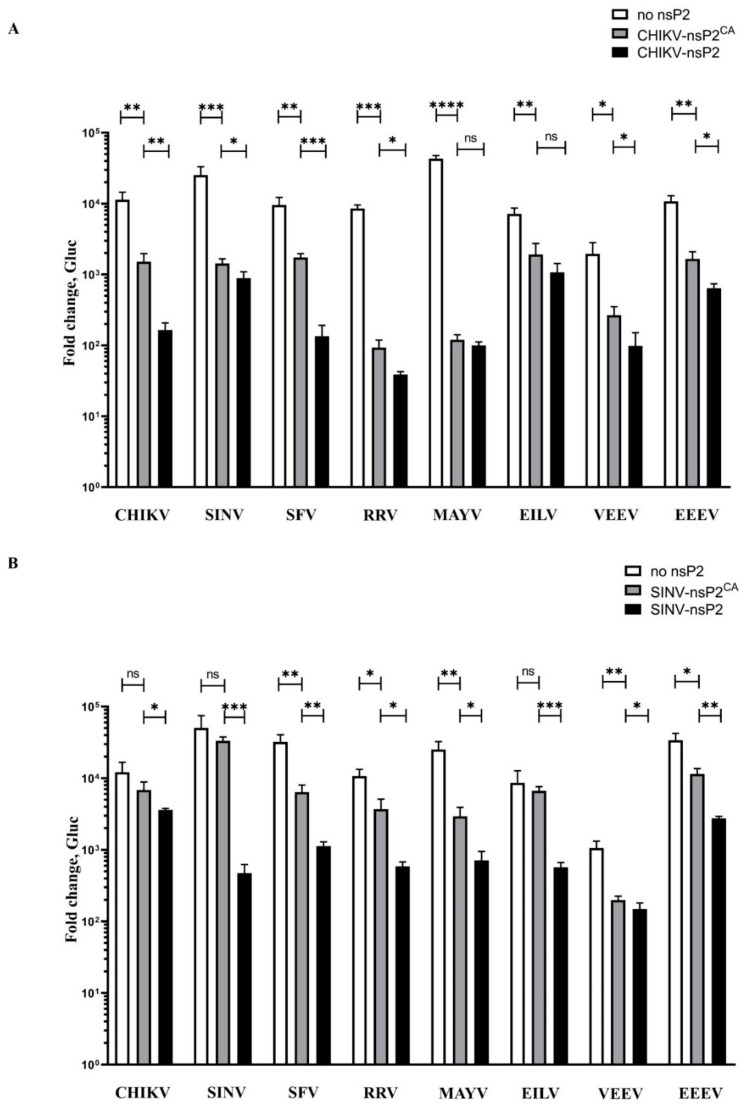
Co-expression of nsP2 of CHIKV or SINV inhibits activity of trans-replicases of heterologous alphaviruses. (**A**) C6/36 cells grown on 96-well plates were co-transfected with matching pairs of AlbPolI-FG and Ubi-P1234 or Ubi-P1234^GAA^ plasmids of alphaviruses shown at X-axes and with pPubi-CHIKV-nsP2^CA^, pPubi-CHIKV-nsP2 or dummy plasmid (no-nsP2 control). Cells were incubated at 28 ^°^C and lysed 48 hpt. Data represent the Gluc activity from Ubi-P1234-CHIKV transfected cells normalized to the Ubi-P1234^GAA^ control cells. Value obtained for P1234^GAA^ control was taken as 1. (**B**). Experiment was performed as described for panel A except that pPubi-SINV-nsP2^CA^ or pPubi-SINV-nsP2 plasmids were used. (**A**,**B**). Means ± SD are shown for three biological replicates. ns, not significant, * *p* < 0.05, ** *p* < 0.01, *** *p* < 0.001, **** *p* < 0.0001 (one-way ANOVA test).

Higher amounts of co-transfected CHIKV nsP2 expression plasmids caused prominent inhibition of CHIKV trans-replicase activity: in this experiment, approximately 6-fold and 73-fold reductions of transcription activities were caused by nsP2^CA^ and wt nsP2, respectively (Figure 4A). Co-expression of CHIKV nsP2^CA^ also strongly reduced activities of trans-replicases of all analyzed arthropod-transmitted alphaviruses; the effect was smallest for SFV (approximately 5.7-fold) and largest for MAYV (approximately 300-fold). No clear correlation between observed inhibition and phylogenetic relationship of viruses was observed. The impact of co-expression of CHIKV nsP2^CA^ on the trans-replicase of insect-specific EILV was smallest, approximately 3.3-fold (Figure 4A). Activities of all trans-replicases were additionally reduced by protease activity of CHIKV nsP2. However, the extent of additional inhibition was much larger (approximately 12-fold) for homologous trans-replicase and that of the closely related SFV (approximately 14-fold) (Figure 4A). It is conceivable that this may reflect the ability of CHIKV nsP2 to process the ns polyprotein of SFV. Protease activity of CHIKV nsP2 had a smaller additional impact on activities of trans-replicases of RRV (approximately 2.3-fold) and MAYV, other members of the SFV complex. The impact of protease activity was also mild on trans-replicases of SINV (approximately 1.5-fold), EILV (approximately 1.7-fold) and New World alphaviruses (approximately 2.5-fold) (Figure 4A). The relatively small differences of inhibition caused by nsP2^CA^ and wt nsP2 of CHIKV on activities of trans-replicases of the majority of heterologous alphaviruses may indicate that they are not (at least not exclusively) caused by protease activity of nsP2. Instead, or in addition, they may originate from some other difference between wt nsP2 and nsP2^CA^, such as the presence of 10 amino acid residues originating from nsP1 at the N-terminus of nsP2^CA^ (due to the lack of protease activity, these residues are not removed unless by protease activity of the nsP2 from the trans-replicase). 

The reduction of trans-replicase activity caused by co-expression of nsP2^CA^ of SINV was less prominent than that caused by co-expression of nsP2^CA^ of CHIKV: the inhibition was from approximately 3-fold for CHIKV to approximately 11-fold for MAYV (Figure 4B). These data indicate that the protease activity-independent dominant negative effect of SINV nsP2^CA^ on RNA replicases of alphaviruses is less prominent than that of CHIKV nsP2^CA^. At the same time, the protease activity of nsP2 more prominently contributed to inhibition of the activity of homologous trans-replicase (approximately 58-fold additional reduction). Surprisingly, the protease activity of SINV nsP2 also strongly affected activity of EILV trans-replicase (approximately 12-fold additional reduction), while 3–6-fold additional reductions were observed for trans-replicases of other viruses, except CHIKV and VEEV, in which cases, the additional impact caused by protease activity of SINV nsP2 was lower (Figure 4B). Overall, the impact of nsP2 of SINV on trans-replicases of different alphaviruses was more dependent on its protease activity. This property possibly reflects ability of the nsP2 of SINV to cleave 2/3 sites in ns polyproteins of different alphaviruses, as has been demonstrated in the case of ns polyprotein of SFV [14].

## 4. Discussion

Numerous positive-strand RNA viruses use their genomic RNA(s) as mRNA(s) for the translation of polyprotein(s) that are precursors for viral replicase proteins. For these viruses, the proteolytic processing of these polyprotein(s) by virus-encoded protease(s) represents an obligatory step in the infection cycle. The processing of the ns polyproteins of alphaviruses by the protease activity of nsP2 is a well-regulated and balanced process; therefore, its disturbance has deleterious impact on alphavirus infection [11,51]. In addition to its protease activity, nsP2 has numerous additional enzymatic activities, is involved in multiple virus-host interactions [7] and has been suggested to have an important role in the interaction between viruses occurring in superinfected cells [39,42]. However, as a protease, alphavirus nsP2 is highly specific, and its cleavages do not only depend on amino acid sequence of the cleavage site but also on its presentation to the protease [58]. Coupled with observations that alphavirus infection can cause SIE for viruses belonging to other virus families [44,59], this indicates that there are nsP2 protease activity-independent mechanisms for SIE in addition to protease-dependent ones.

In this study, we found clear evidence that, in *Aedes albopictus* C6/36 cells, co-expressed nsP2 and its protease activity can inhibit formation and/or activities of RNA replicase of the same or other alphaviruses. The data from experiments performed using trans-replicase assays and VRP infection were highly similar. Interestingly, while the protease activity of co-expressed nsP2 was clearly important for reduction of RNA synthesis by trans-replicases, mutations that are known to increase this activity did not, at least directly, prominently increase ability of nsP2 of CHIKV (Figure 2B) or SINV (Figure 2C) to inhibit activities of corresponding RNA replicases. The most likely explanation is that the Asn614 to Asp mutation present in nsP2^ND^ of SINV did not increase its activity toward critical 2/3 site cleavage, which depends mostly on the presentation of the site to the protease [14]. This is in agreement with our previous findings demonstrating that hyper-activating mutation in SINV nsP2^ND^ acts via enhanced and/or premature cleavage of the 1/2 site [11] that for alphaviruses occurs in cis [15,60]. It can be speculated that the same also applies for mutations introduced into CHIKV nsP2^EV^. We also did not observe that mutations designed to alter the sub-cellular localization of co-expressed nsP2 had major impact on its ability to interfere with RNA replicase formation/activity (Figure 2A,B). Most likely, this indicates that a substantial fraction of co-expressed wt nsP2 was localized in the cytoplasm of co-transfected cells and was capable of interfering with RNA replicase formation/activity; therefore, an increase of amount of cytoplasmic nsP2 (at the expense of the nuclear fraction) did not result in additional inhibition. 

While our data about the importance of nsP2 for an effect that mimics SIE is in agreement with several previous reports [39,42], it is different from observations made in a more recent study [44]. There are several possible explanations why the authors of the latter study failed to observe a role of nsP2 in the block of viral replication and SIE. The strategy used for expression of nsP2 protein was different: here we produced nsP2 with its correct N-terminal region; such an enzyme is fully capable for cleavage of all three sites in ns polyprotein. In the other study, an nsP2 with an extra N-terminal Met-residue was expressed. Although seemingly minor, such an extension has drastic impact on protease activity of nsP2. In particular, a single extra amino acid residue at the N-terminus of nsP2 almost completely abolishes the ability of nsP2 SFV to process the 2/3 site in the ns polyprotein [14], i.e., prevents the most important event needed to suppress formation of active RNA replicase (Figure 2B). While this technical difference serves as the most likely explanation why the role of nsP2 protease remained undetected in study by Boussier et al. [44], it is less clear why, in contrast to our study, nsP2 protease activity independent inhibition was not seen. It is possible that this was due to different experimental conditions including use of mammalian rather than mosquito cells, different expression strategies and levels of nsP2 as well as methods used to detect the inhibition of RNA replication. However, it cannot be excluded that some protease-independent effects detected in this study are also consequences of our expression strategy. The addition of 10 amino acid residues from the C-terminus of nsP1 to the sequence of nsP2 allowed the generation of correct N-terminus of nsP2 only when the nsP2 had protease activity. In the case of nsP2^CA^, these residues were therefore not removed resulting in protein with extra 10 amino acid residues. The consequences of this extension on non-protease activities of nsP2 are unknown. It has been demonstrated that nsP2 with N-terminal EGFP was also able to interfere with interferon signaling and exhibited cytotoxic effects in mammalian cells [61]. An N-terminal affinity tag of comparable length to our 10 amino acid nsP1 extension did not eliminate any of enzymatic activities associated with N-terminal domains of nsP2; only some modulation of NTPase, RNA helicase and RNA annealing abilities were observed [25]. Therefore, we consider it unlikely that the N-terminal extension of nsP2^CA^ substantially influence the observed effects.

It was observed that co-expression of nsP2 of CHIKV or SINV reduced the activity of trans-replicases of heterologous virus as well as these of homologous alphaviruses. Interestingly, for CHIKV nsP2, the effect was mostly protease activity-independent, i.e., co-expression of CHIKV nsP2^CA^ strongly reduced the activity of trans-replicases of all arboviruses included in this study (Figure 4A). The protease activity increased the negative effect from co-expression of CHIKV nsP2 but, with the exception of trans-replicases of CHIKV and related SFV, the additional impact caused by protease activity was mild or, for trans-replicases of MAYV and SINV, almost undetectable. These data most likely reflect the ability of CHIKV nsP2 to cleave the ns polyprotein of SFV at the 2/3 site and its inability to do the same with ns polyprotein of SINV, since this is the case for nsP2 of the related SFV [14]. Determining whether the mild additional reductions of activity of trans-replicases of RRV, EEEV, VEEV and EILV are also due to the cleavage of their ns polyproteins by nsP2 of CHIKV requires specific analysis that was beyond the scope of the current study. In contrast to nsP2 of CHIKV, co-expression of nsP2 of SINV caused more prominent protease activity-dependent reduction of activities of trans-replicases of heterologous alphaviruses (Figure 4B). The effect was significant, albeit small, even for CHIKV, an observation that differs from that made using VRP infection (compare Figure 3 and Figure 4B). Most likely, this discrepancy results from the different amounts of protease expression plasmid used in these experiments, which in trans-replicase experiments was approximately 5-fold higher (normalized to the number of transfected cells). Interestingly, protease activity of nsP2 of SINV strongly affected the activity of the trans-replicase of insect-specific EILV; this finding probably indicates an ability of nsP2 of SINV to efficiently cleave ns polyprotein of EILV. It has also been previously observed that infection by EILV induces strong SIE for SINV, reducing its titers up to 10,000 fold. EILV infection was also found to reduce titers of other alphaviruses, the effect being smallest—10- to 52-fold—for EEEV and CHIKV [40]. Interestingly, an impact of protease activity of nsP2 of CHIKV on trans-replicase of EILV was also clearly smaller than in the case of nsP2 of SINV (Figure 4A,B). These data suggest a closer relationship between RNA replicases of SINV and EILV an assumption that is supported by finding that trans-replicases of these viruses are able to cross-utilize each other’s template RNAs [46]. 

Based primarily on the use of trans-replicase based assays, it was revealed that co-expression of nsP2 of CHIKV and SINV interferes with formation and/or functioning of RNA replicase of homologous and heterologous alphaviruses. This property was observed for protease-dead nsP2 mutants, but significantly stronger in wild-type (protease-active) nsP2, indicating both protease-dependent and protease-independent effects. Using CHIKV ns polyproteins with mutations in the 1/2 or 2/3 cleavage site revealed that cleavage at the latter site is crucial for inhibition of RNA replication. These findings are all consistent with a hypothesis that nsP2 and its protease activity play important roles in SIE in mosquito cells. However, it should be acknowledged that, although trans-replicase represents a convenient model, it does not precisely reproduce the conditions of a real superinfection event. It also remains unclear whether the effects of nsP2 co-expression in vertebrate cells are similar to these observed in mosquito cells. Such experiments were outside of scope of the current study and may not produce unambiguous data due to the very high cytotoxicity of nsP2 of Old World alphaviruses for vertebrate cells. Still, even with these limitations, alphavirus trans-replicases can be used as tools for studies of interaction between RNA replicases and co-expressed proteins important for RNA replication, including host cell factors. Such studies could provide better understanding of the multiple roles of virus-encoded proteins and host cell proteins in the formation and functioning of alphavirus RCs. This knowledge also has the potential to be used to develop novel approaches to inhibit alphavirus replication. Thus, our data indicates that expression of nsP2 in mosquitoes can generate resistance against not only homologous alphavirus but a panel of different alphaviruses pathogenic to humans. 

## Data Availability

All data is present on figures, raw data is presented as Supplementary Materials.

## Supplementary Materials

The following supporting information can be downloaded at: https://www.mdpi.com/article/10.3390/v14061327/s1: Table S1: Results of trans-replicase assay; File S1: Results of FACS analysis; File S2: original images of western blots.

## References

[1] Chen R., Mukhopadhyay S., Merits A., Bolling B., Nasar F., Coffey L.L., Powers A., Weaver S.C. (2019). ICTV Report Consortium ICTV Virus Taxonomy Profile: Togaviridae. J. Gen. Virol..

[2] Nasar F., Palacios G., Gorchakov R.V., Guzman H., Da Rosa A.P., Savji N., Popov V.L., Sherman M.B., Lipkin W.I., Tesh R.B. (2012). Eilat virus, a unique alphavirus with host range restricted to insects by RNA replication. Proc. Natl. Acad. Sci. USA.

[3] Fros J.J., Pijlman G.P. (2016). Alphavirus infection: Host cell shut-off and inhibition of antiviral responses. Viruses.

[4] Vega-Rúa A., Zouache K., Girod R., Failloux A.B., Lourenço-de-Oliveira R. (2014). High level of vector competence of Aedes aegypti and Aedes albopictus from ten American countries as a crucial factor in the spread of chikungunya virus. J. Virol..

[5] Burt F.J., Rolph M.S., Rulli N.E., Mahalingam S., Heise M.T. (2012). Chikungunya: A Re-emerging Virus. Lancet.

[6] Amraoui F., Failloux A.B. (2016). Chikungunya: An unexpected emergence in Europe. Curr. Opin. Virol..

[7] Ahola T., McInerney G., Merits A. (2021). Alphavirus RNA replication in vertebrate cells. Adv. Virus Res..

[8] Rausalu K., Utt A., Quirin T., Varghese F.S., Žusinaite E., Das P.K., Ahola T., Merits A. (2016). Chikungunya virus infectivity, RNA replication and non-structural polyprotein processing depend on the nsP2 protease’s active site cysteine residue. Sci. Rep..

[9] Ding M.X., Schlesinger M.J. (1989). Evidence that Sindbis virus NSP2 is an autoprotease which processes the virus nonstructural polyprotein. Virology.

[10] Vasiljeva L., Valmu L., Kääriäinen L., Merits A. (2001). Site-specific Protease Activity of the Carboxyl-terminal Domain of Semliki Forest Virus Replicase Protein nsP2. J. Biol. Chem..

[11] Lulla V., Karo-Astover L., Rausalu K., Saul S., Merits A., Lulla A. (2018). Timeliness of Proteolytic Events Is Prerequisite for Efficient Functioning of the Alphaviral Replicase. J. Virol..

[12] De Groot R.J., Hardy W.R., Shirako Y., Strauss J.H. (1990). Cleavage-site preferences of Sindbis virus polyproteins containing the non-structural proteinase. Evidence for temporal regulation of polyprotein processing in vivo. EMBO J..

[13] Lemm J.A., Rice C.M. (1993). Roles of nonstructural polyproteins and cleavage products in regulating Sindbis virus RNA replication and transcription. J. Virol..

[14] Lulla A., Lulla V., Merits A. (2012). Macromolecular assembly-driven processing of the 2/3 cleavage site in the alphavirus replicase polyprotein. J. Virol..

[15] Vasiljeva L., Merits A., Golubtsov A., Sizemskaja V., Kääriäinen L., Ahola T. (2003). Regulation of the sequential processing of Semliki Forest virus replicase polyprotein. J. Biol. Chem..

[16] Lemm J.A., Rümenapf T., Strauss E.G., Strauss J.H., Rice M.C. (1994). Polypeptide requirements for assembly of functional Sindbis virus replication complexes: A model for the temporal regulation of minus- and plus-strand RNA synthesis. EMBO J..

[17] Hellstrom K., Kallio K., Utt A., Quirin T., Jokitalo E., Merits A., Ahola T. (2017). Partially uncleaved alphavirus replicase forms spherule structures in the presence and absence of RNA template. J. Virol..

[18] Cancedda R., Villa-Komaroff L., Lodish H.F., Schlesinger M. (1975). Initiation sites for translation of Sindbis virus 42S and 26S messenger RNAs. Cell.

[19] Rupp J.C., Sokoloski K.J., Gebhart N.N., Hardy R.W. (2015). Alphavirus RNA synthesis and non-structural protein functions. J. Gen. Virol..

[20] Sawicki D.L., Sawicki S.G. (1980). Short-lived minus-strand polymerase for Semliki Forest virus. J. Virol..

[21] Law Y.S., Utt A., Tan Y.B., Zheng J., Wang S., Chen M.W., Griffin P.R., Merits A., Luo D. (2019). Structural insights into RNA recognition by the Chikungunya virus nsP2 helicase. Proc. Natl. Acad. Sci. USA.

[22] Rikkonen M., Peränen J., Kääriänen L. (1994). ATPase and GTPase activities associated with Semliki Forest virus nonstructural protein nsP2. J. Virol..

[23] Vasiljeva L., Merits A., Auvinen P., Kääriäinen L. (2000). Identification of a novel function of the alphavirus capping apparatus: RNA 5’-triphosphatase activity of nsp2. J. Biol. Chem..

[24] De Cedron M.G., Ehsani N., Mikkola M., Garcia J.A. (1999). RNA helicase activity of Semliki Forest virus replicase protein nsP2. FEBS Lett..

[25] Das P.K., Merits A., Lulla A. (2014). Functional cross-talk between distant domains of chikungunya virus non-structural protein 2 is decisive for its RNA-modulating activity. J. Biol. Chem..

[26] Russo A.T., White M.A., Watowich S.J. (2006). The crystal structure of the Venezuelan equine encephalitis alphavirus nsP2 protease. Structure.

[27] Law Y.S., Wang S., Tan Y.B., Shih O., Utt A., Goh W.Y., Lian B.J., Chen M.W., Jeng U.S., Merits A. (2021). Interdomain Flexibility of Chikungunya Virus nsP2 Helicase-Protease Differentially Influences Viral RNA Replication and Infectivity. J. Virol..

[28] Peränen J., Rikkonen M., Liljeström P., Kääriäinen L. (1990). Nuclear localization of Semliki Forest virus-specific nonstructural protein nsP2. J. Virol..

[29] Akhrymuk I., Kulemzin S.V., Frolova E.I. (2012). Evasion of Innate Immune Response: The Old World Alphavirus nsP2 Protein Induces Rapid Degradation of Rpb1, a Catalytic Subunit of RNA Polymerase II. J. Virol..

[30] Fros J.J., Liu W.J., Prow N.A., Geertsema C., Ligtenberg M., Vanlandingham D.L., Schnettler E., Vlak J.M., Suhrbier A., Khromykh A.A. (2010). Chikungunya Virus Nonstructural Protein 2 Inhibits Type I/II Interferon-Stimulated JAK-STAT Signaling. J. Virol..

[31] Goertz G.P., McNally K.L., Robertson S.J., Best S.M., Pijlman G.P., Fros J.J. (2018). The methyltransferase-like domain of chikungunya virus nsP2 inhibits the interferon response by promoting the nuclear export of STAT1. J. Virol..

[32] Tamm K., Merits A., Sarand I. (2008). Mutations in the nuclear localization signal of nsP2 influencing RNA synthesis, protein expression and cytotoxicity of Semliki Forest virus. J. Gen. Virol..

[33] Perri S., Driver D.A., Gardner J.P., Sherrill S., Belli B.A., Dubensky T.W., Polo J.M. (2000). Replicon vectors derived from Sindbis virus and Semliki forest virus that establish persistent replication in host cells. J. Virol..

[34] Utt A., Das P.K., Varjak M., Lulla V., Lulla A., Merits A. (2015). Mutations conferring a noncytotoxic phenotype on chikungunya virus replicons compromise enzymatic properties of nonstructural protein 2. J. Virol..

[35] Akhrymuk I., Lukash T., Frolov I. (2019). Novel Mutations in nsP2 Abolish Chikungunya Virus-Induced Transcriptional Shutoff and Make the Virus Less Cytopathic without Affecting Its Replication Rates. J. Virol..

[36] Akhrymuk I., Frolov I., Frolova E.I. (2018). Sindbis Virus Infection Causes Cell Death by nsP2-Induced Transcriptional Shutoff or by nsP3-Dependent Translational Shutoff. J. Virol..

[37] Stollar V., Shenk T.E. (1973). Homologous viral interference in Aedes albopictus cultures chronically infected with Sindbis virus. J. Virol..

[38] Eaton B.T. (1979). Heterologous interference in Aedes albopictus cells infected with alphaviruses. J. Virol..

[39] Karpf A.R., Lenches E., Strauss E.G., Strauss J.H., Brown D.T. (1997). Superinfection exclusion of alphaviruses in three mosquito cell lines persistently infected with Sindbis virus. J. Virol..

[40] Nasar F., Erasmus J.H., Haddow A.D., Tesh R.B., Weaver S.C. (2015). Eilat virus induces both homologous and heterologous interference. Virology.

[41] Adams R.H., Brown D.T. (1985). BHK cells expressing Sindbis virus induced homologous interference allow the translation of nonstructural genes of superinfecting virus. J. Virol..

[42] Sawicki D.L., Perri S., Polo J.M., Sawicki S.G. (2006). Role for nsP2 proteins in the cessation of alphavirus minus-strand synthesis by host cells. J. Virol..

[43] Ehrengruber M.U., Goldin A.L. (2007). Semliki Forest virus vectors with mutations in the nonstructural protein 2 gene permit extended superinfection of neuronal and non-neuronal cells. J. Neurovirol..

[44] Boussier J., Levi L., Weger-Lucarelli J., Poirier E.Z., Vignuzzi M., Albert M.L. (2020). Chikungunya virus superinfection exclusion is mediated by a block in viral replication and does not rely on non-structural protein 2. PLoS ONE.

[45] Singer Z.S., Ambrose P.M., Danino T., Rice C.M. (2021). Quantitative measurements of early alphaviral replication dynamics in single cells reveals the basis for superinfection exclusion. Cell Syst..

[46] Lello L.S., Utt A., Bartholomeeusen K., Wang S., Rausalu K., Kendall C., Coppens S., Fragkoudis R., Tuplin A., Alphey L. (2020). Cross-utilisation of template RNAs by alphavirus replicases. PLoS Pathog..

[47] Bartholomeeusen K., Utt A., Coppens S., Rausalu K., Vereecken K., Ariën K.K., Merits A. (2018). A Chikungunya Virus trans -Replicase System Reveals the Importance of Delayed Nonstructural Polyprotein Processing for Efficient Replication Complex Formation in Mosquito Cells. J. Virol..

[48] Utt A., Rausalu K., Jakobson M., Männik A., Alphey L., Fragkoudis R., Merits A. (2019). Design and Use of Chikungunya Virus Replication Templates Utilizing Mammalian and Mosquito RNA Polymerase I-Mediated Transcription. J. Virol..

[49] Glasker S., Lulla A., Lulla V., Couderc T., Drexler J.F., Liljeström P., Lecuit M., Drosten C., Merits A., Kümmerer B.M. (2013). Virus replicon particle-based Chikungunya virus neutralization assay using Gaussia luciferase as readout. Virol. J..

[50] Rikkonen M., Peranen J., Kaariainen L. (1992). Nuclear and nucleolar targeting signals of Semliki Forest virus nonstructural protein nsP2. Virology.

[51] Strauss E.G., De Groot R.J., Levinson R., Strauss J.H. (1992). Identification of the active site residues in the nsP2 proteinase of Sindbis virus. Virology.

[52] Spuul P., Balistreri G., Hellström K., Golubtsov A.V., Jokitalo E., Ahola T. (2011). Assembly of alphavirus replication complexes from RNA and protein components in a novel trans-replication system in mammalian cells. J. Virol..

[53] Kallio K., Hellström K., Balistreri G., Spuul P., Jokitalo E., Ahola T. (2013). Template RNA length determines the size of replication complex spherules for Semliki Forest virus. J. Virol..

[54] Abraham R., Hauer D., McPherson R.L., Utt A., Kirby I.T., Cohen M.S., Merits A., Leung A.K.L., Griffin D.E. (2018). ADP-ribosyl-binding and hydrolase activities of the alphavirus nsP3 macrodomain are critical for initiation of virus replication. Proc. Natl. Acad. Sci. USA.

[55] Lello L.S., Bartholomeeusen K., Wang S., Coppens S., Fragkoudis R., Alphey L., Arien K.K., Merits A., Utt A. (2021). nsP4 is a major determinant of alphavirus replicase activity and template selectivity. J. Virol..

[56] Saul S., Ferguson M., Cordonin C., Fragkoudis R., Ool M., Tamberg N., Sherwood K., Fazakerley J.K., Merits A. (2015). Differences in Processing Determinants of Nonstructural Polyprotein and in the Sequence of Nonstructural Protein 3 Affect Neurovirulence of Semliki Forest Virus. J. Virol..

[57] Suopanki J., Sawicki D.L., Sawicki S.G., Kaariainen L. (1998). Regulation of alphavirus 26S mRNA transcription by replicase component nsP2. J. Gen. Virol..

[58] Lulla V., Karo-Astover L., Rausalu K., Merits A., Lulla A. (2013). Presentation Overrides Specificity: Probing the Plasticity of Alphaviral Proteolytic Activity through Mutational Analysis. J. Virol..

[59] Muturi E.J., Bara J. (2015). Sindbis virus interferes with dengue 4 virus replication and its potential transmission by Aedes albopictus. Parasites Vectors.

[60] Hardy W.R., Strauss J.H. (1989). Processing the nonstructural polyproteins of Sindbis virus: Nonstructural proteinase is in the C-terminal half of nsP2 and functions both in cis and in trans. J. Virol..

[61] Fros J.J., Van der Maten E., Vlak J.M., Pijlman G.P. (2013). The C-terminal domain of chikungunya virus nsP2 independently governs viral RNA replication, cytopathicity and inhibition of interferon signaling. J. Virol..

