# Supplementary material for: Expression of Alphavirus Nonstructural Protein 2 (nsP2) in Mosquito Cells Inhibits Viral RNA Replication in Both a Protease Activity-Dependent and -Independent Manner
Source: Viruses. 2022 Jun 17;14(6):1327. doi: 10.3390/v14061327 (PMC9228716; doi:10.3390/v14061327)
Supplement: Supplementary file 1 [file viruses-14-01327-s001.zip › File S1 Results of FACS analysis/CHIKV nsP2 infected with CHIKV VRPs.pdf]

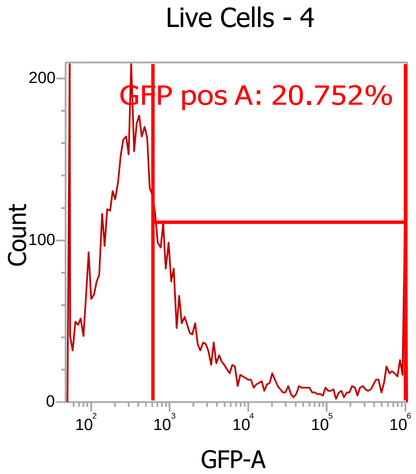


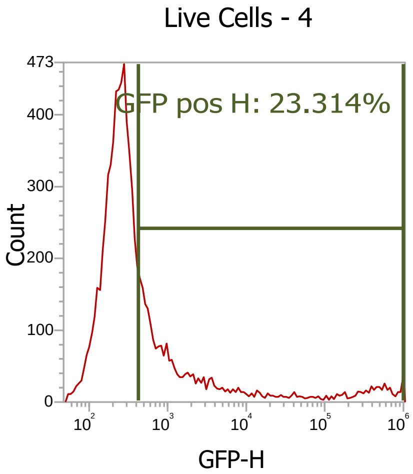


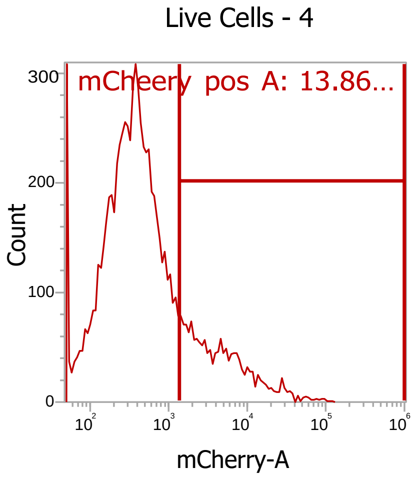


Experiment: Ljuba 22.11.2021
Sample: CHIKV nsP2 + VRPs
Time Recorded: 12:23:26

| Name | Gate | Count | %Total | %Gated | X Mean | X Media |
|---|---|---|---|---|---|---|
| All Events | All Events | 11,671 | 100.000 | 100.000 | N/A | N/A |
| Live Cells | Live Cells | 8,435 | 72.273 | 72.273 | 294,910 | 280,71 |
| GFP pos H | GFP pos H | 2,721 | 23.314 | 32.258 | 78,358 | 1,250 |
| GFP pos A | GFP pos A | 2,422 | 20.752 | 28.714 | 125,260 | 2,155 |
| mCheery pos A | mCheery pos A | 1,618 | 13.863 | 19.182 | 8,067 | 4,116 |
| mCheery pos H | mCheery pos H | 1,716 | 14.703 | 20.344 | 6,595 | 3,195 |
| GFP-/mCherry+ | GFP-/mCherry+ | 774 | 6.632 | 9.176 | 60 | 131 |
| GFP+/mCherry+ | GFP+/mCherry+ | 119 | 1.020 | 1.411 | 99,989 | 2,954 |
| GFP-/mCherry- | GFP-/mCherry- | 5,816 | 49.833 | 68.951 | 109 | 157 |
| GFP+/mCherry- | GFP+/mCherry- | 1,726 | 14.789 | 20.462 | 168,622 | 4,452 |

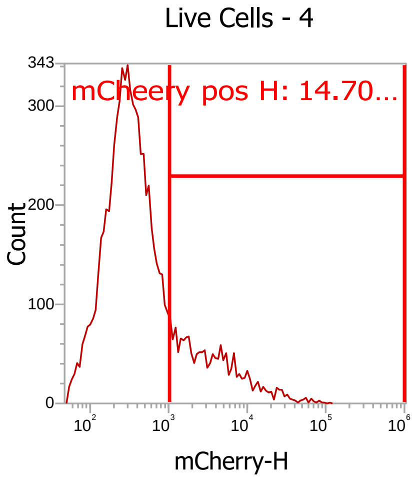


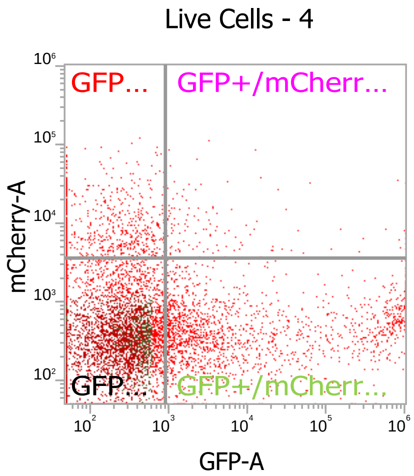

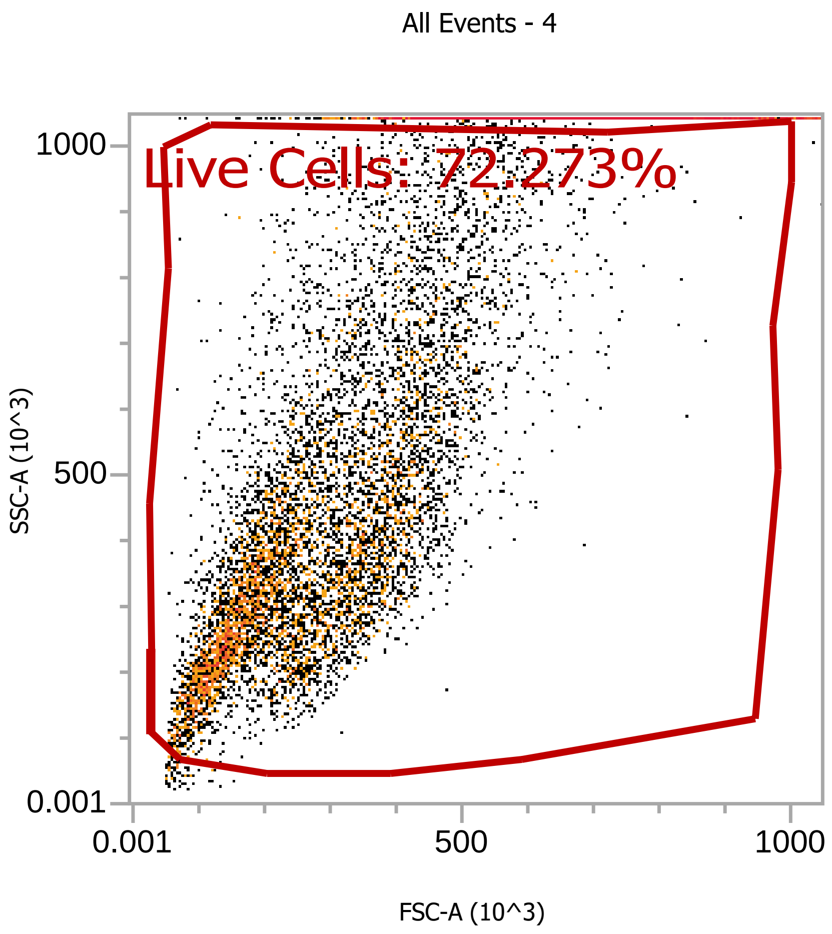
All Events - 4
Live Cells: 72.273%
SSC-A (10^3)
1000
500
0.001
0.001
500
1000
FSC-A (10^3)

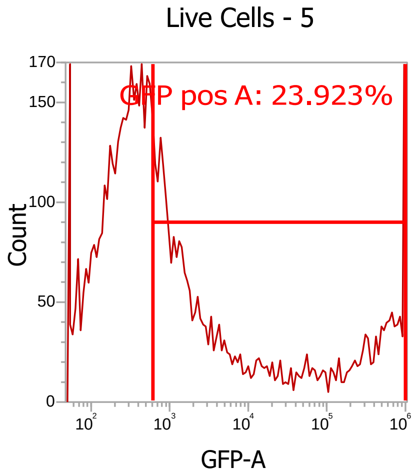


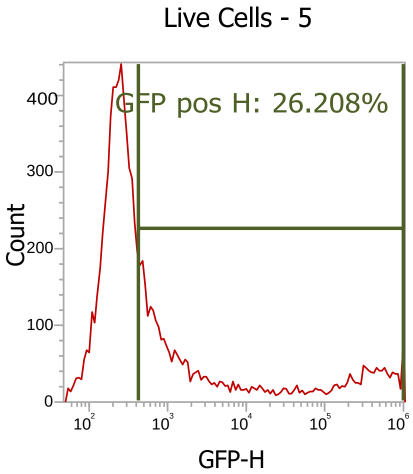


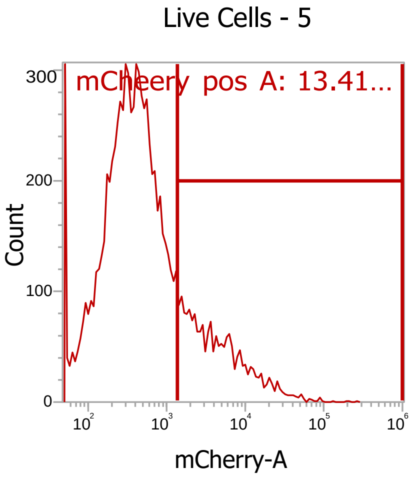


Experiment: Ljuba 22.11.2021
Sample: CHIKV nsP2 + VRPs
Time Recorded: 12:27:48

| Name | Gate | Count | %Total | %Gated | X Mean | X Media |
|---|---|---|---|---|---|---|
| All Events | All Events | 14,442 | 100.000 | 100.000 | N/A | N/A |
| Live Cells | Live Cells | 9,561 | 66.203 | 66.203 | 326,287 | 328,62 |
| GFP pos H | GFP pos H | 3,785 | 26.208 | 39.588 | 126,669 | 2,237 |
| GFP pos A | GFP pos A | 3,455 | 23.923 | 36.136 | 198,804 | 4,380 |
| mCheery pos A | mCheery pos A | 1,938 | 13.419 | 20.270 | 7,597 | 3,819 |
| mCheery pos H | mCheery pos H | 2,081 | 14.409 | 21.766 | 6,207 | 3,019 |
| GFP-/mCherry+ | GFP-/mCherry+ | 830 | 5.747 | 8.681 | 34 | 116 |
| GFP+/mCherry+ | GFP+/mCherry+ | 218 | 1.509 | 2.280 | 156,684 | 2,863 |
| GFP-/mCherry- | GFP-/mCherry- | 5,931 | 41.068 | 62.033 | 53 | 135 |
| GFP+/mCherry- | GFP+/mCherry- | 2,582 | 17.878 | 27.006 | 252,598 | 18,541 |

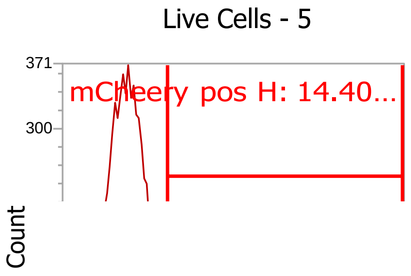


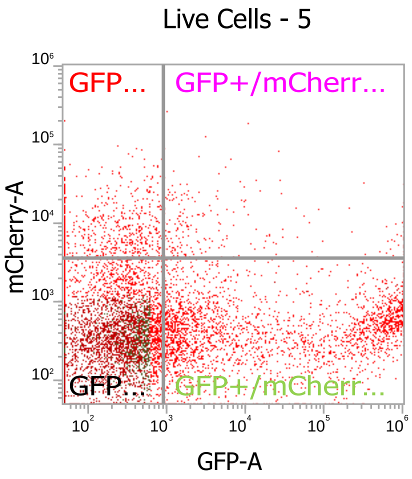

All Events - 5

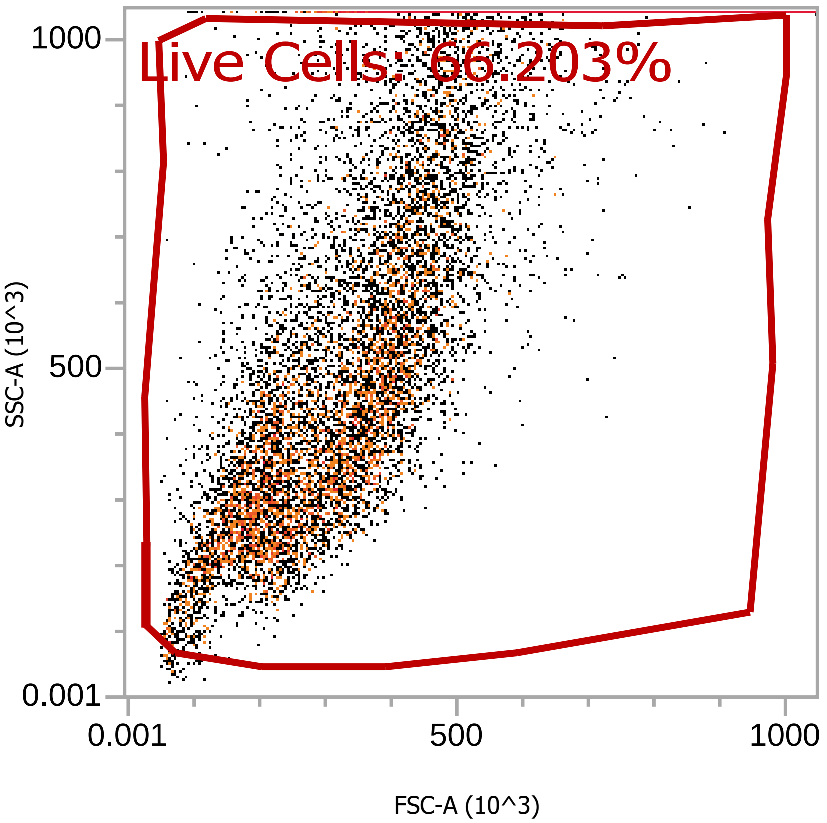

Live Cells: 66.203%
1000
500
0.001
SSC-A (10^3)
0.001
500
1000
FSC-A (10^3)

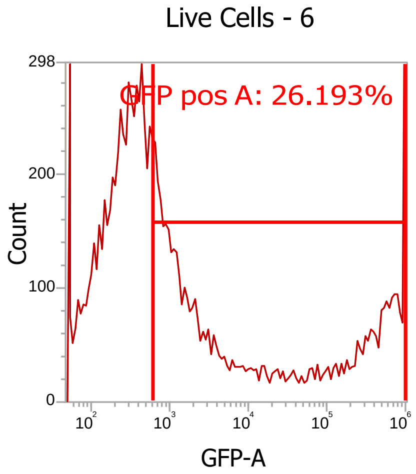


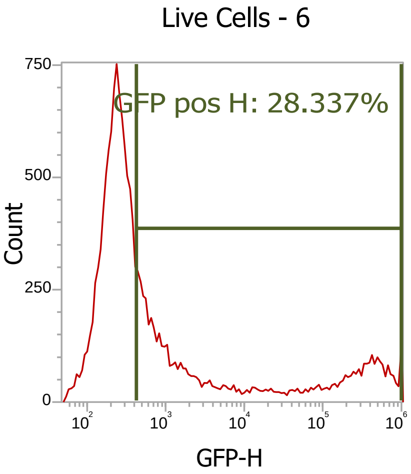


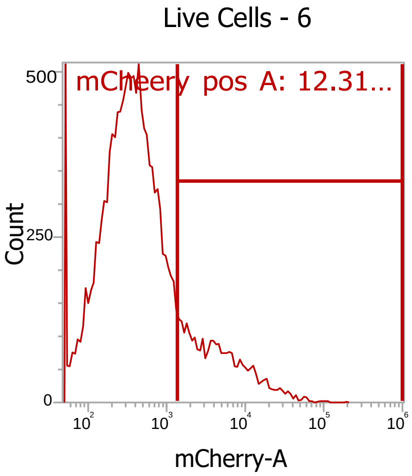


Experiment: Ljuba 22.11.2021
Sample: CHIKV nsP2 + VRPs
Time Recorded: 12:32:10

| Name | Gate | Count | %Total | %Gated | X Mean | X Media |
|---|---|---|---|---|---|---|
| All Events | All Events | 22,525 | 100.000 | 100.000 | N/A | N/A |
| Live Cells | Live Cells | 15,727 | 69.820 | 69.820 | 346,662 | 355,13 |
| GFP pos H | GFP pos H | 6,383 | 28.337 | 40.586 | 141,206 | 3,110 |
| GFP pos A | GFP pos A | 5,900 | 26.193 | 37.515 | 221,701 | 6,959 |
| mCheery pos A | mCheery pos A | 2,774 | 12.315 | 17.638 | 7,804 | 4,172 |
| mCheery pos H | mCheery pos H | 2,973 | 13.199 | 18.904 | 6,357 | 3,375 |
| GFP-/mCherry+ | GFP-/mCherry+ | 1,288 | 5.718 | 8.190 | 54 | 110 |
| GFP+/mCherry+ | GFP+/mCherry+ | 306 | 1.358 | 1.946 | 143,463 | 2,890 |
| GFP-/mCherry- | GFP-/mCherry- | 9,583 | 42.544 | 60.933 | 49 | 141 |
| GFP+/mCherry- | GFP+/mCherry- | 4,550 | 20.200 | 28.931 | 277,659 | 41,682 |

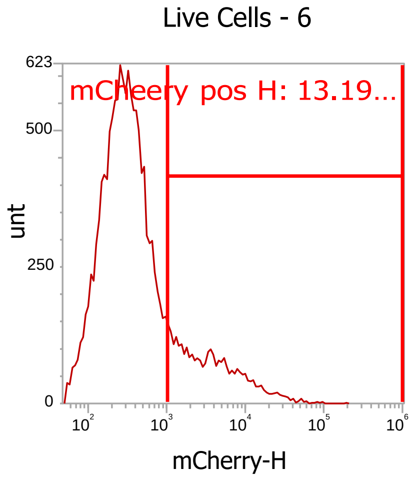


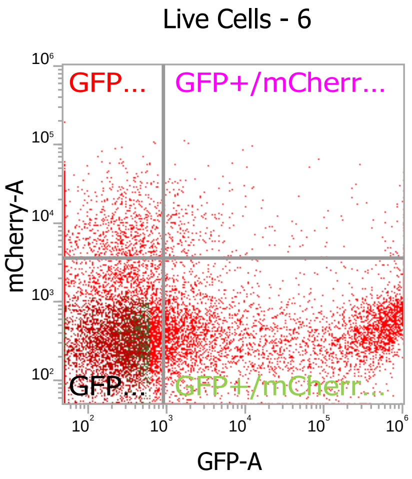

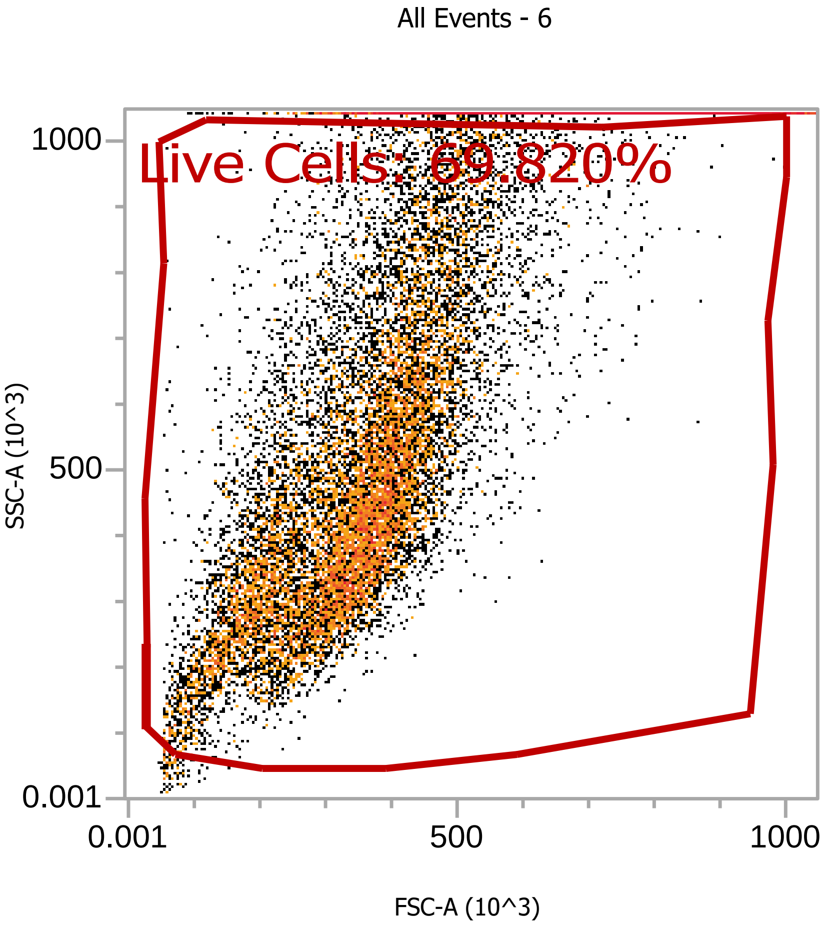
All Events - 6
Live Cells: 69.820%
1000
500
0.001
SSC-A (10^3)
0.001
500
1000
FSC-A (10^3)
