# Supplementary material for: Expression of Alphavirus Nonstructural Protein 2 (nsP2) in Mosquito Cells Inhibits Viral RNA Replication in Both a Protease Activity-Dependent and -Independent Manner
Source: Viruses. 2022 Jun 17;14(6):1327. doi: 10.3390/v14061327 (PMC9228716; doi:10.3390/v14061327)
Supplement: Supplementary file 1 [file viruses-14-01327-s001.zip › File S1 Results of FACS analysis/CHIKV nsP2CA infected with CHIKV VRPs.pdf]

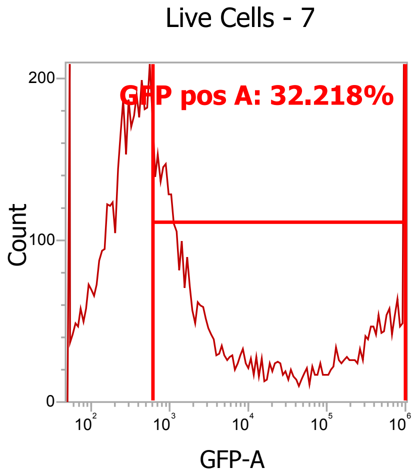

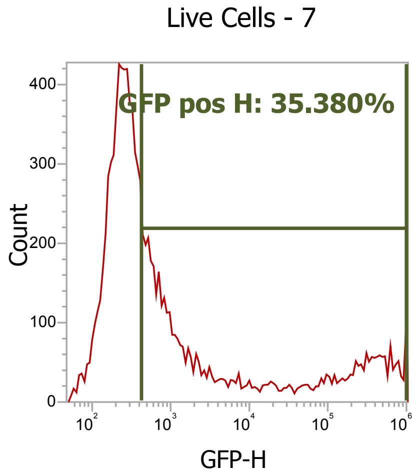

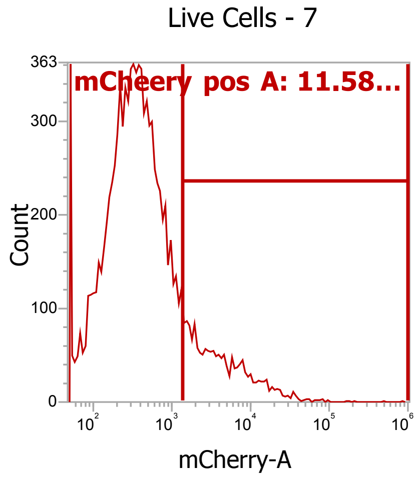

Experiment:

Sample: CHIKV nsP2CA+VRPs

Time Recorded: **12:36:33**

Name	Gate	Count	%Total	%Gated	X Mean	X Media
All Events	All Events	14,169	100.000	100.000	N/A	N/A
Live Cells	Live Cells	10,897	76.907	76.907	331,591	337,19
GFP pos H	GFP pos H	5,013	35.380	46.003	128,793	2,275
GFP pos A	GFP pos A	4,565	32.218	41.892	203,390	4,948
mCheery pos A	mCheery pos A	1,642	11.589	15.068	8,035	3,644
mCheery pos H	mCheery pos H	1,753	12.372	16.087	6,471	2,871
GFP-/mCherry+	GFP-/mCherry+	586	4.136	5.378	76	141
GFP+/mCherry+	GFP+/mCherry+	265	1.870	2.432	304,849	10,001
GFP-/mCherry-	GFP-/mCherry-	6,563	46.319	60.228	99	175
GFP+/mCherry-	GFP+/mCherry-	3,483	24.582	31.963	243,199	19,18[illegible]

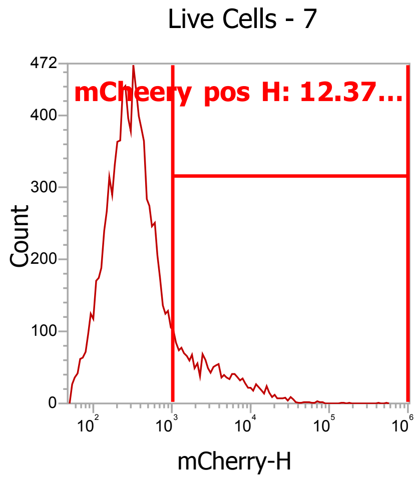

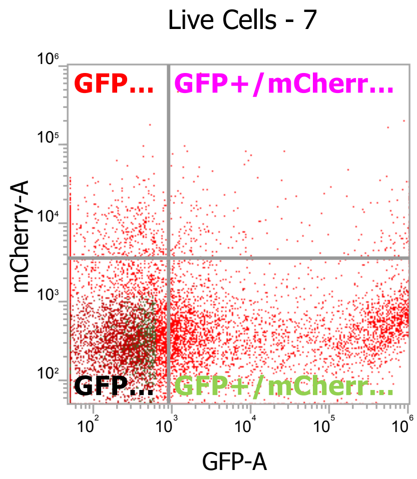

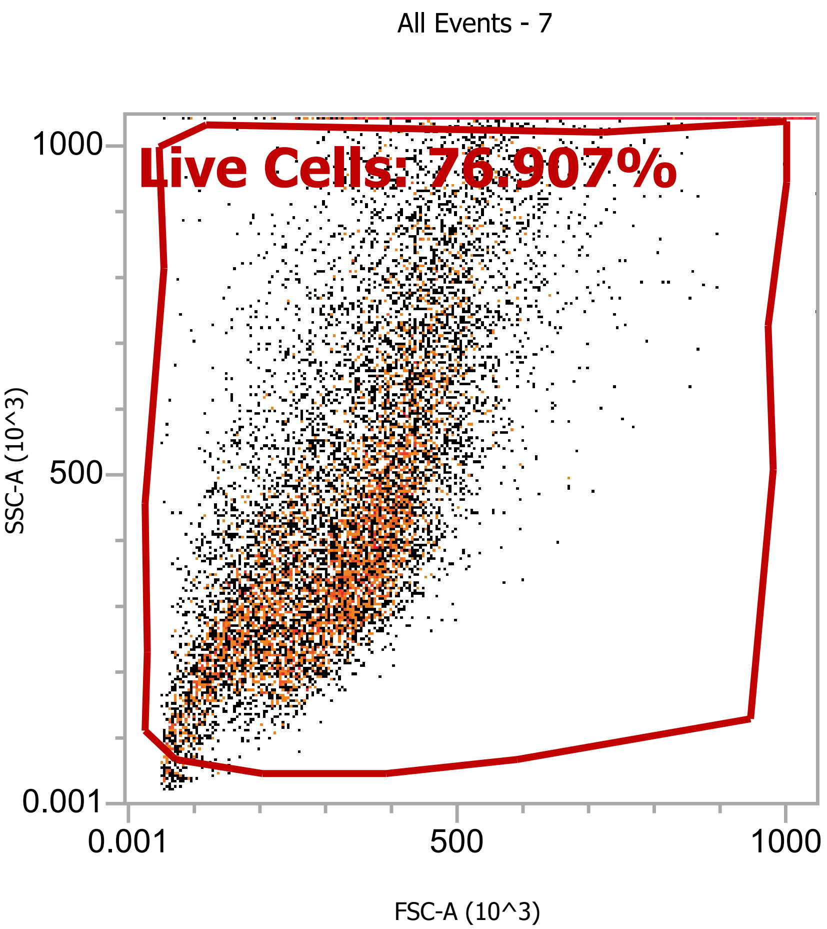
All Events - 7
Live Cells: 76.907%
1000
500
0.001
SSC-A (10^3)
0.001
500
1000
FSC-A (10^3)

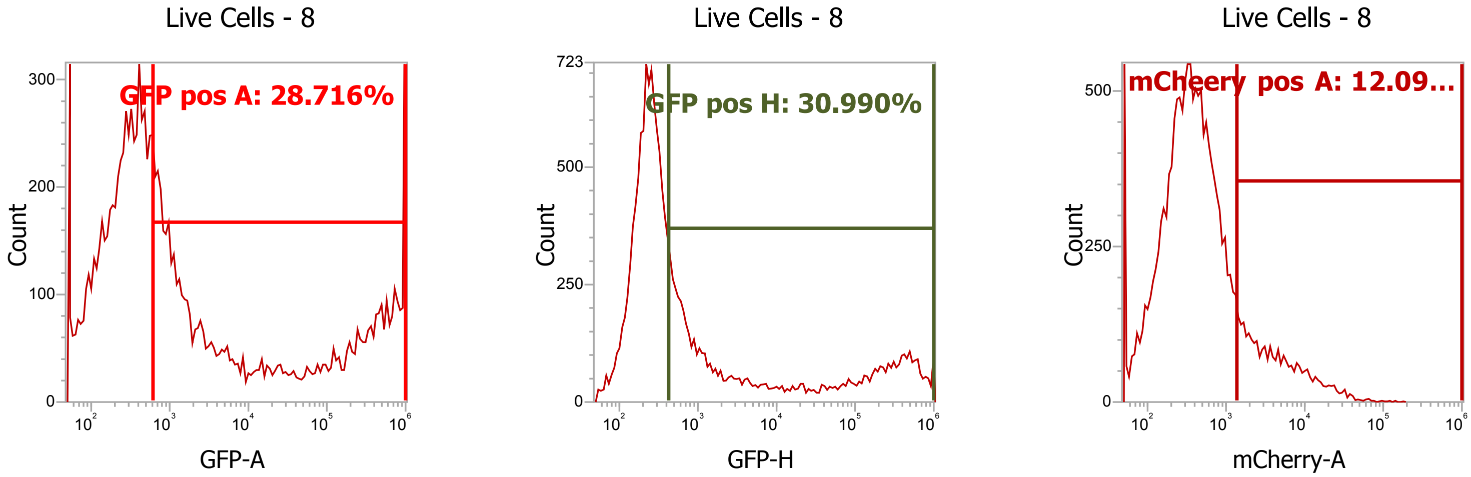

Experiment:
Sample: CHIKV nsP2CA+VRPs
Time Recorded: **12:40:58**

Name	Gate	Count	%Total	%Gated	X Mean	X Media
All Events	All Events	21,946	100.000	100.000	N/A	N/A
Live Cells	Live Cells	16,225	73.931	73.931	343,044	349,97
GFP pos H	GFP pos H	6,801	30.990	41.917	135,695	4,373
GFP pos A	GFP pos A	6,302	28.716	38.841	216,504	10,423
mCheery pos A	mCheery pos A	2,655	12.098	16.364	7,522	3,815
mCheery pos H	mCheery pos H	2,849	12.982	17.559	6,114	2,980
GFP-/mCherry+	GFP-/mCherry+	1,019	4.643	6.280	23	129
GFP+/mCherry+	GFP+/mCherry+	393	1.791	2.422	310,452	35,082
GFP-/mCherry-	GFP-/mCherry-	9,978	45.466	61.498	37	137
GFP+/mCherry-	GFP+/mCherry-	4,835	22.031	29.800	256,791	44,521

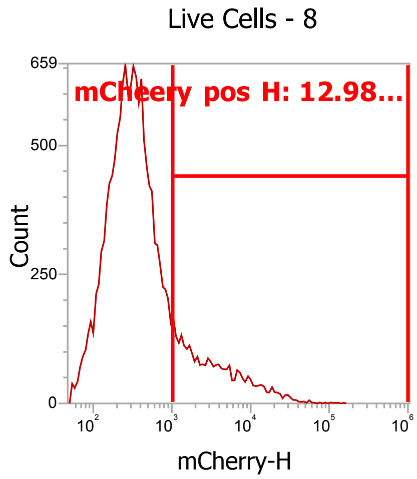

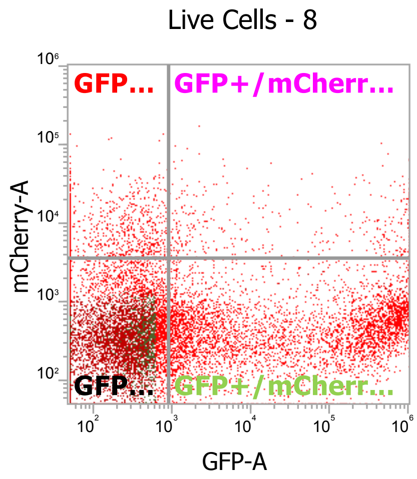

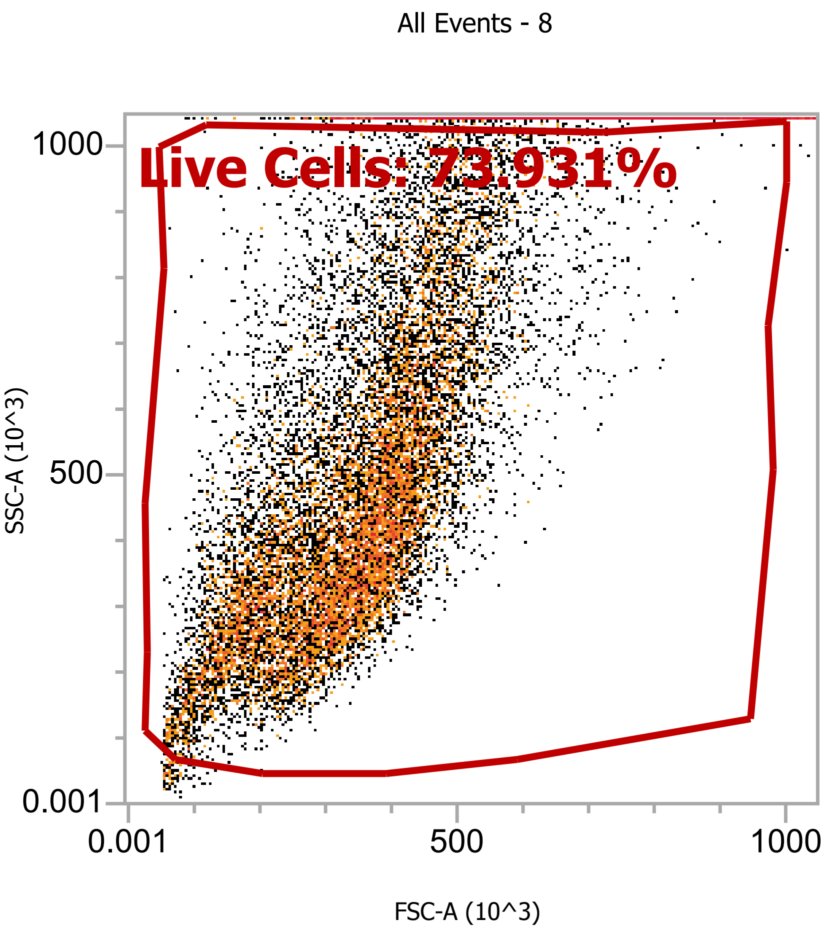

All Events - 8
Live Cells: 73.931%
1000
500
0.001
SSC-A (10^3)
0.001
500
1000
FSC-A (10^3)

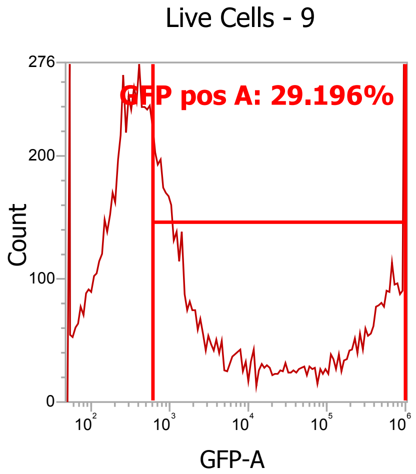

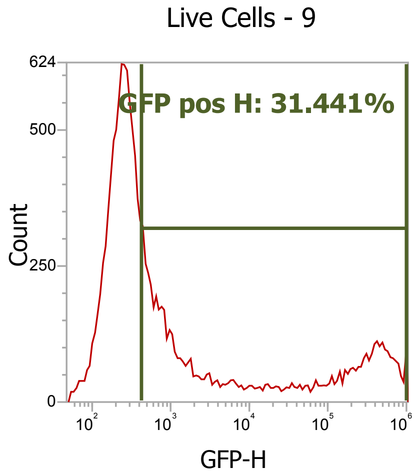

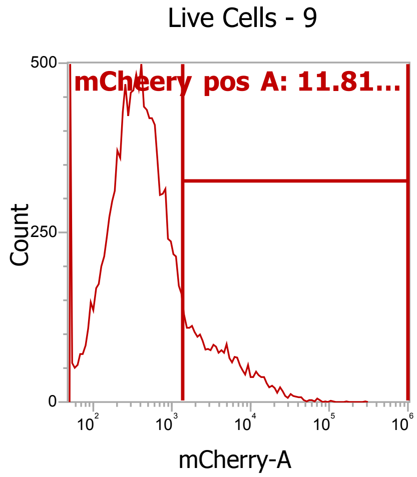

Experiment:
Sample: CHIKV nsP2CA+VRPs
Time Recorded: **12:45:23**

Name	Gate	Count	%Total	%Gated	X Mean	X Media
All Events	All Events	21,246	100.000	100.000	N/A	N/A
Live Cells	Live Cells	14,911	70.183	70.183	345,559	354,44
GFP pos H	GFP pos H	6,680	31.441	44.799	144,801	3,991
GFP pos A	GFP pos A	6,203	29.196	41.600	230,786	9,035
mCheery pos A	mCheery pos A	2,511	11.819	16.840	7,033	3,736
mCheery pos H	mCheery pos H	2,726	12.831	18.282	5,693	2,905
GFP-/mCherry+	GFP-/mCherry+	979	4.608	6.566	106	137
GFP+/mCherry+	GFP+/mCherry+	355	1.671	2.381	338,587	57,516
GFP-/mCherry-	GFP-/mCherry-	8,792	41.382	58.963	68	165
GFP+/mCherry-	GFP+/mCherry-	4,785	22.522	32.090	273,888	42,340

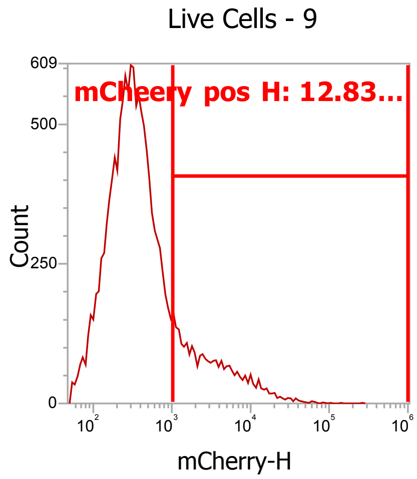

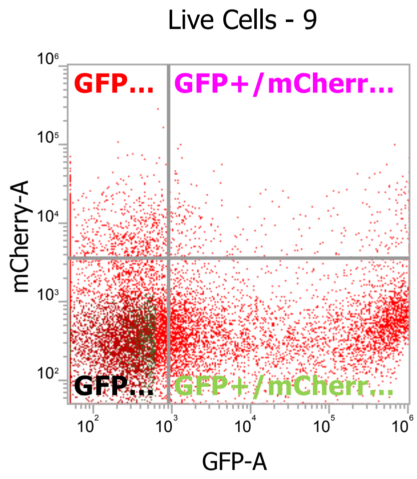

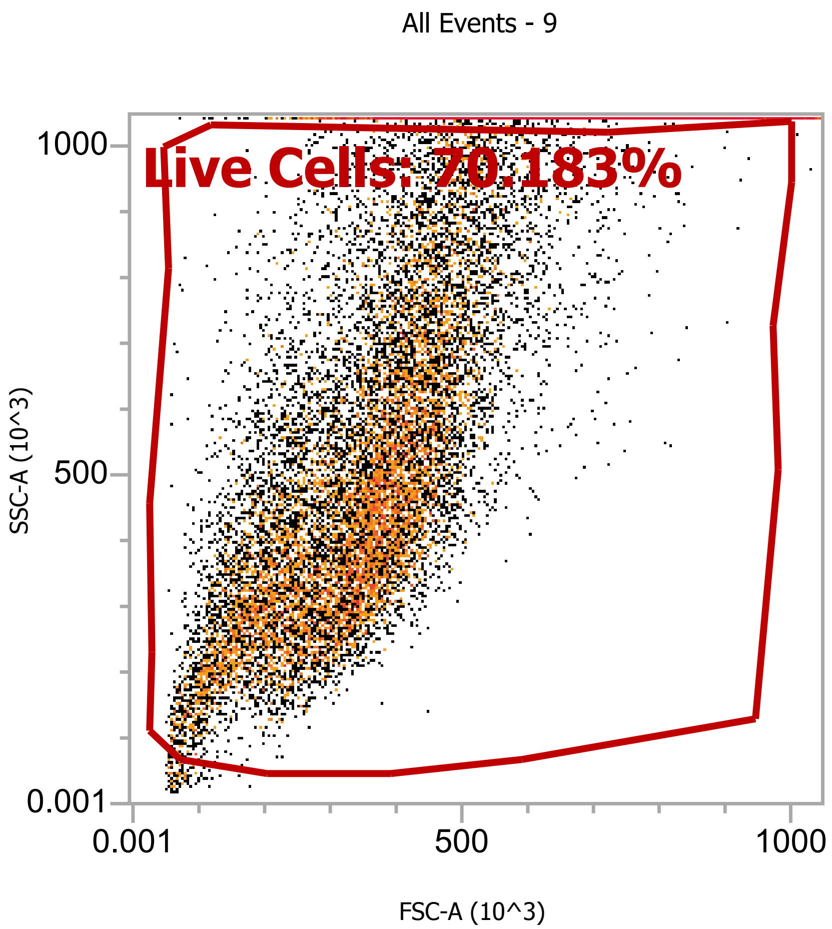
All Events - 9
Live Cells: 70.183%
1000
500
0.001
SSC-A (10^3)
0.001
500
1000
FSC-A (10^3)
