# Supplementary material for: Expression of Alphavirus Nonstructural Protein 2 (nsP2) in Mosquito Cells Inhibits Viral RNA Replication in Both a Protease Activity-Dependent and -Independent Manner
Source: Viruses. 2022 Jun 17;14(6):1327. doi: 10.3390/v14061327 (PMC9228716; doi:10.3390/v14061327)
Supplement: Supplementary file 1 [file viruses-14-01327-s001.zip › File S1 Results of FACS analysis/Control transfection with DsRed plasmid and infected with CHIKV VRPs (1).pdf]

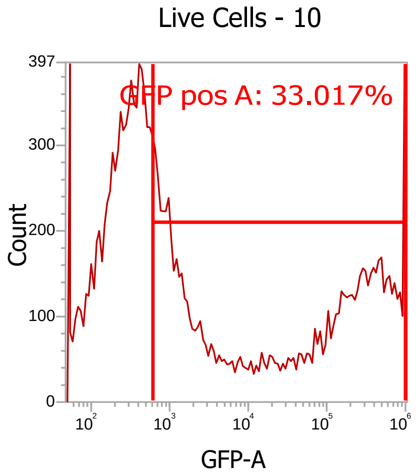

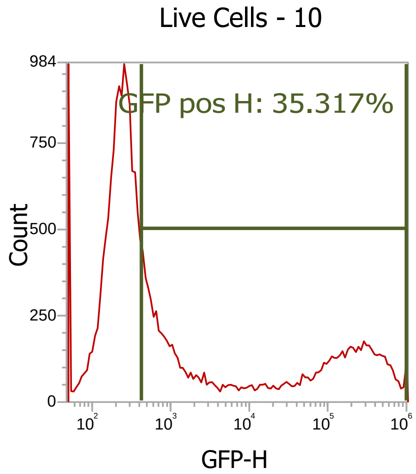

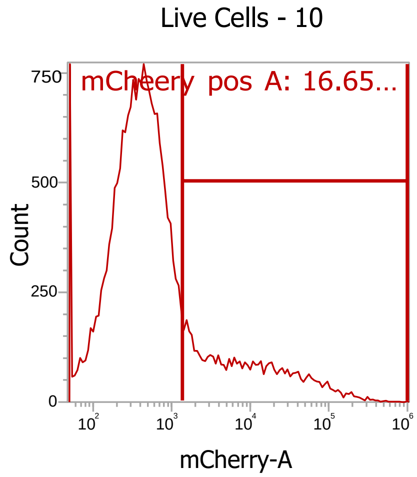

Experiment: Ljuba mcherry and gfp
Sample: 10
Time Recorded: 14:03:55

Name	Gate	Count	%Total	%Gated	X Mean	X Media
All Events	All Events	30,000	100.000	100.000	N/A	N/A
Live Cells	Live Cells	23,509	78.363	78.363	374,851	370,25
GFP pos H	GFP pos H	10,595	35.317	45.068	135,203	16,689
GFP pos A	GFP pos A	9,905	33.017	42.133	214,173	46,200
mCheery pos A	mCheery pos A	4,997	16.657	21.256	27,421	8,083
mCheery pos H	mCheery pos H	5,112	17.040	21.745	23,321	6,602
GFP-/mCherry+	GFP-/mCherry+	1,978	6.593	8.414	23	184
GFP+/mCherry+	GFP+/mCherry+	1,484	4.947	6.312	288,128	163,79
GFP-/mCherry-	GFP-/mCherry-	13,044	43.480	55.485	12	127
GFP+/mCherry-	GFP+/mCherry-	7,003	23.343	29.789	241,714	99,517

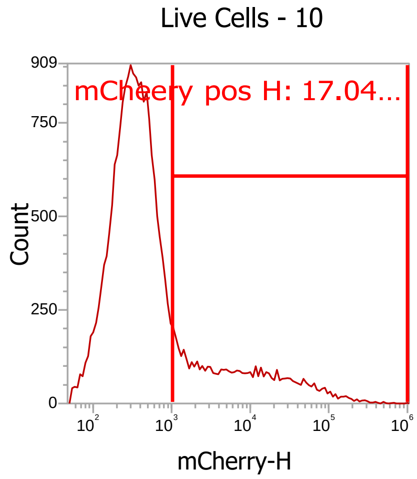

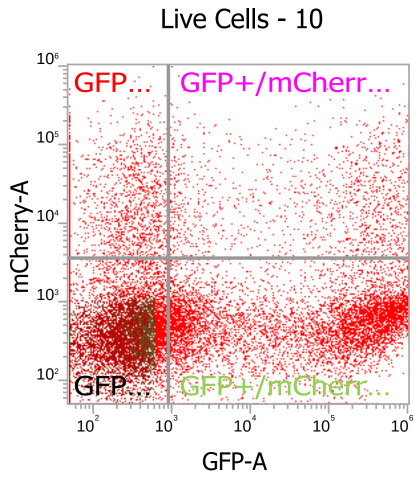

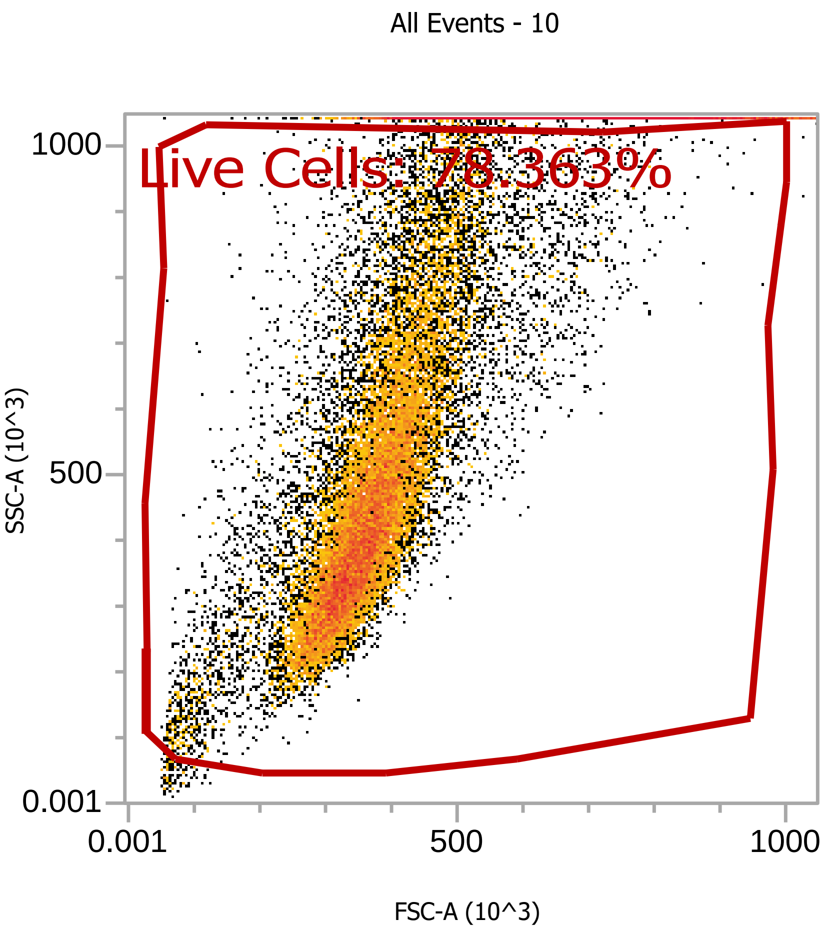
All Events - 10
Live Cells: 78.363%
1000
500
0.001
SSC-A (10^3)
0.001
500
1000
FSC-A (10^3)

Live Cells - 12

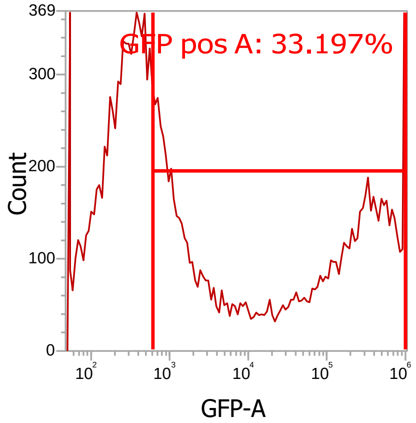

Live Cells - 12

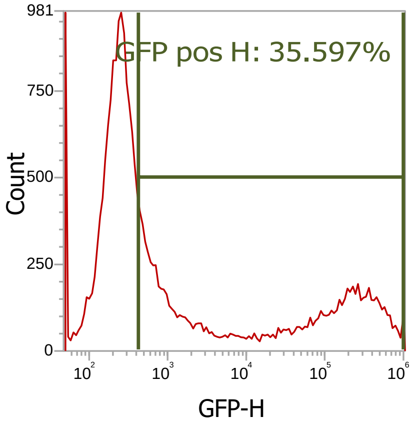

Live Cells - 12

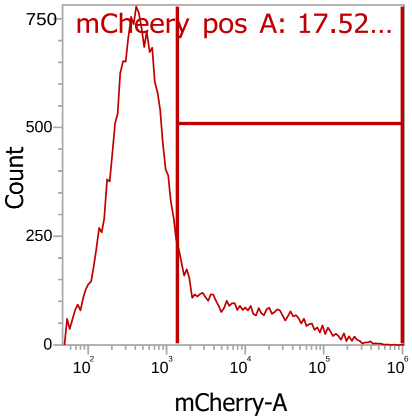

Experiment: Ljuba mcherry and gfp
Sample: 12
Time Recorded: 14:08:00

Name	Gate	Count	%Total	%Gated	X Mean	X Media
All Events	All Events	30,000	100.000	100.000	N/A	N/A
Live Cells	Live Cells	23,389	77.963	77.963	375,025	370,17
GFP pos H	GFP pos H	10,679	35.597	45.658	139,173	20,229
GFP pos A	GFP pos A	9,959	33.197	42.580	221,529	52,257
mCheery pos A	mCheery pos A	5,257	17.523	22.476	27,051	7,328
mCheery pos H	mCheery pos H	5,335	17.783	22.810	23,039	6,029
GFP-/mCherry+	GFP-/mCherry+	2,002	6.673	8.560	36	159
GFP+/mCherry+	GFP+/mCherry+	1,548	5.160	6.618	284,357	168,24
GFP-/mCherry-	GFP-/mCherry-	12,809	42.697	54.765	-1	122
GFP+/mCherry-	GFP+/mCherry-	7,030	23.433	30.057	251,062	100,78

Live Cells - 12

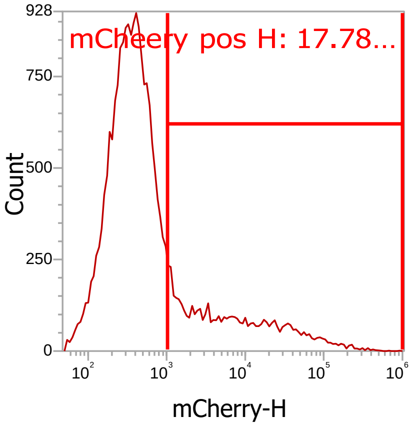

Live Cells - 12

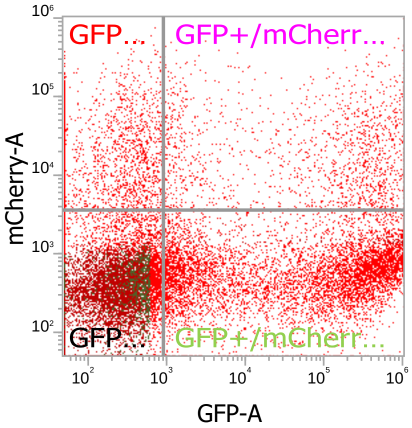

All Events - 12

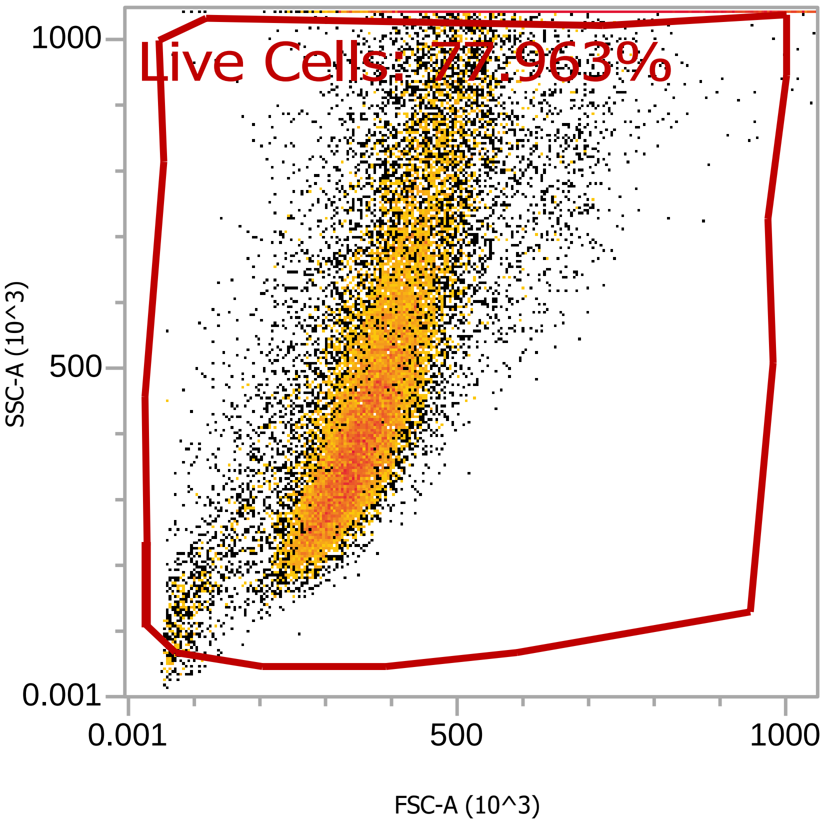

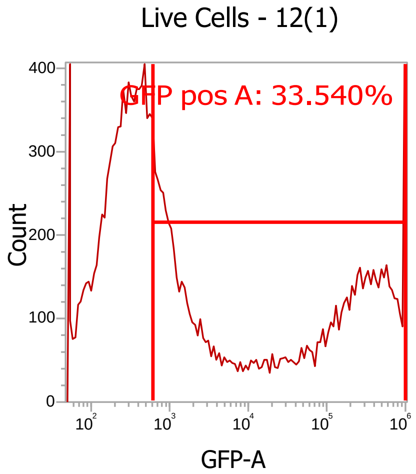

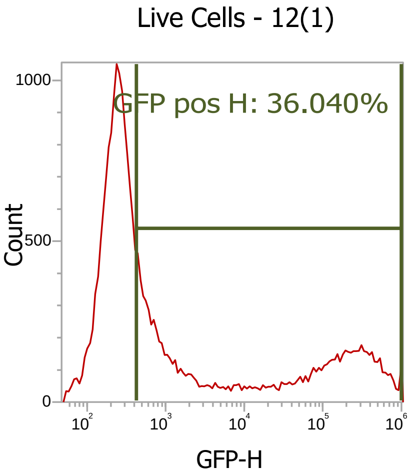

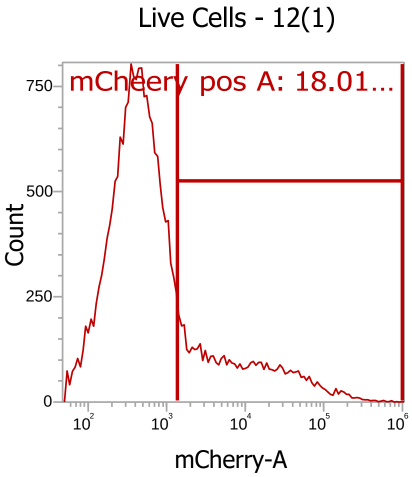

Experiment: Ljuba mcherry and gfp
Sample: 12(1)
Time Recorded: 14:11:08

Name	Gate	Count	%Total	%Gated	X Mean	X Media
All Events	All Events	30,000	100.000	100.000	N/A	N/A
Live Cells	Live Cells	24,216	80.720	80.720	370,643	366,09
GFP pos H	GFP pos H	10,812	36.040	44.648	132,464	14,531
GFP pos A	GFP pos A	10,062	33.540	41.551	210,070	40,512
mCheery pos A	mCheery pos A	5,403	18.010	22.312	27,177	7,852
mCheery pos H	mCheery pos H	5,521	18.403	22.799	23,130	6,418
GFP-/mCherry+	GFP-/mCherry+	2,212	7.373	9.134	84	215
GFP+/mCherry+	GFP+/mCherry+	1,509	5.030	6.231	277,449	151,97
GFP-/mCherry-	GFP-/mCherry-	13,414	44.713	55.393	33	133
GFP+/mCherry-	GFP+/mCherry-	7,081	23.603	29.241	239,222	90,370

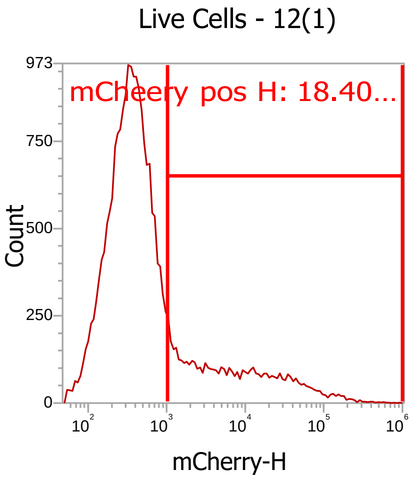

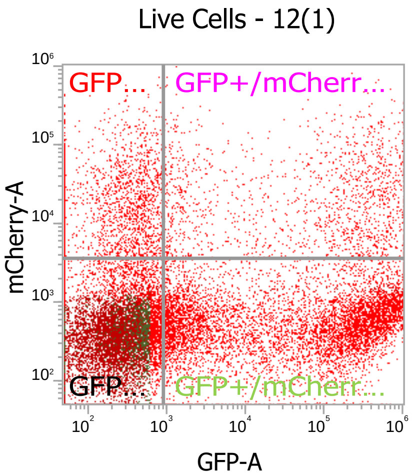

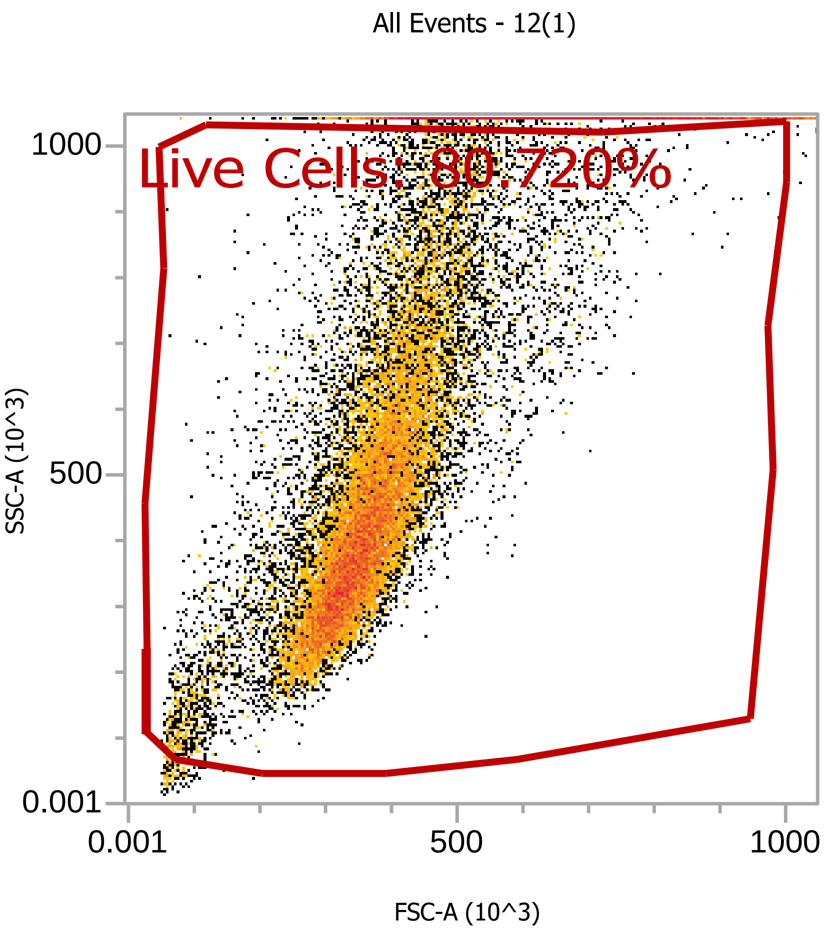
All Events - 12(1)
Live Cells: 80.720%
1000
500
0.001
SSC-A (10^3)
0.001
500
1000
FSC-A (10^3)
