# Supplementary material for: Expression of Alphavirus Nonstructural Protein 2 (nsP2) in Mosquito Cells Inhibits Viral RNA Replication in Both a Protease Activity-Dependent and -Independent Manner
Source: Viruses. 2022 Jun 17;14(6):1327. doi: 10.3390/v14061327 (PMC9228716; doi:10.3390/v14061327)
Supplement: Supplementary file 1 [file viruses-14-01327-s001.zip › File S1 Results of FACS analysis/SINV nsP2 infected with CHIKV VRPs.pdf]

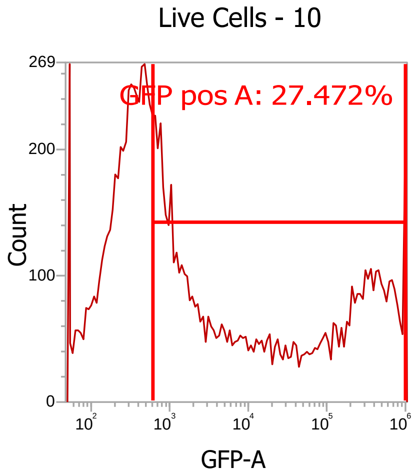

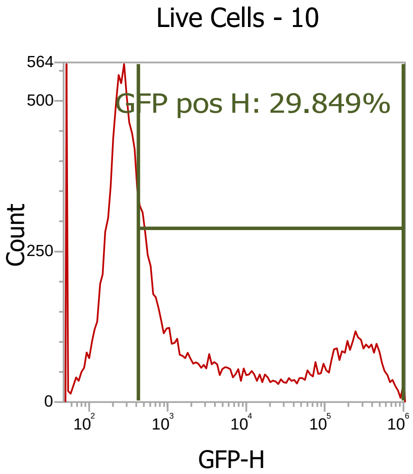

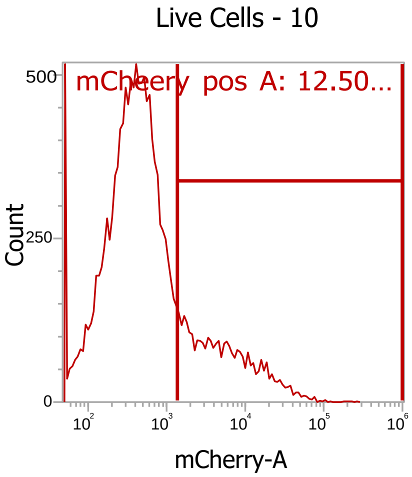

Experiment: Ljuba 22.11.2021
Sample: SINV nsP2 + VRPs
Time Recorded: 12:49:51

Name	Gate	Count	%Total	%Gated	X Mean	X Media
All Events	All Events	25,910	100.000	100.000	N/A	N/A
Live Cells	Live Cells	15,572	60.100	60.100	305,757	311,18
GFP pos H	GFP pos H	7,734	29.849	49.666	98,977	4,931
GFP pos A	GFP pos A	7,118	27.472	45.710	166,909	13,594
mCheery pos A	mCheery pos A	3,239	12.501	20.800	9,418	4,783
mCheery pos H	mCheery pos H	3,380	13.045	21.706	7,903	3,872
GFP-/mCherry+	GFP-/mCherry+	1,387	5.353	8.907	-165	116
GFP+/mCherry+	GFP+/mCherry+	586	2.262	3.763	204,126	41,756
GFP-/mCherry-	GFP-/mCherry-	8,168	31.525	52.453	-103	174
GFP+/mCherry-	GFP+/mCherry-	5,431	20.961	34.877	196,577	36,923

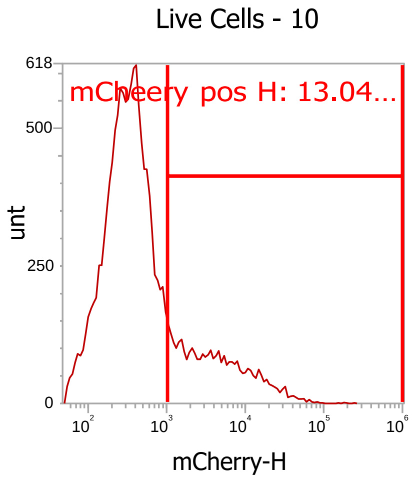

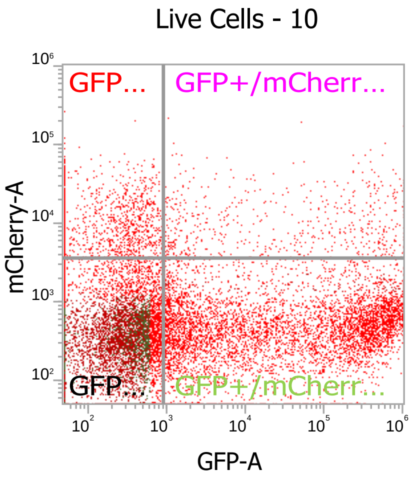

All Events - 10

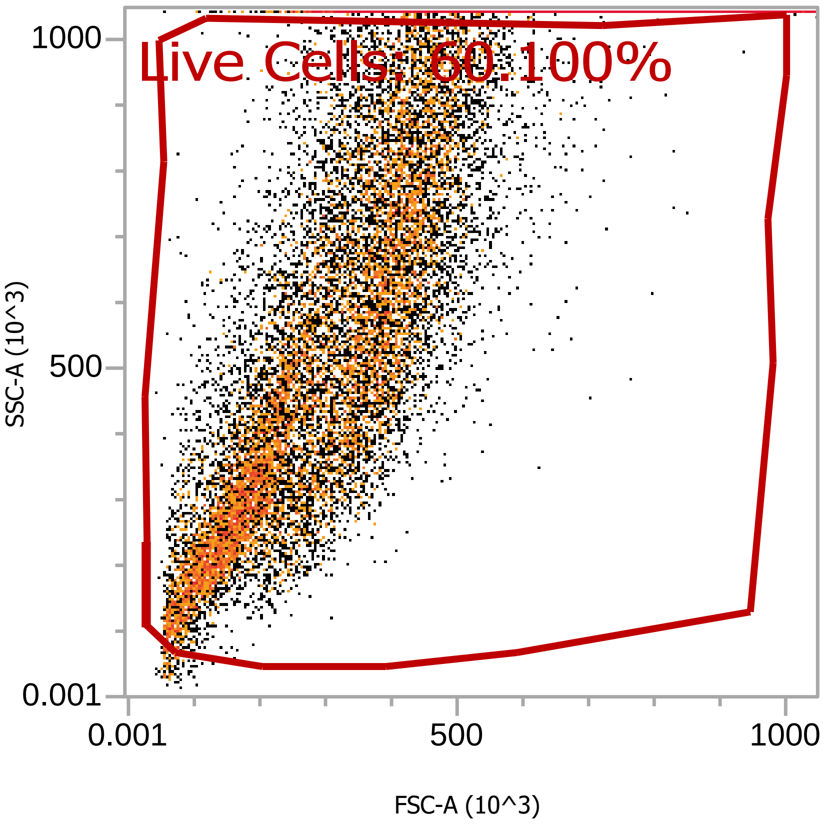

Live Cells - 11

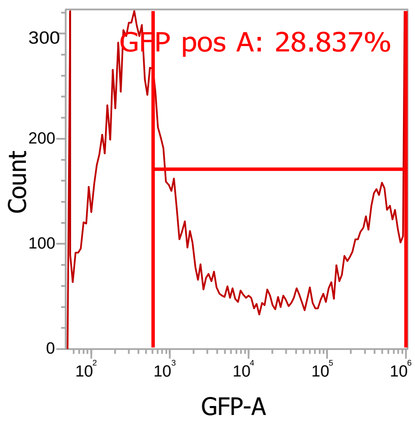

Live Cells - 11

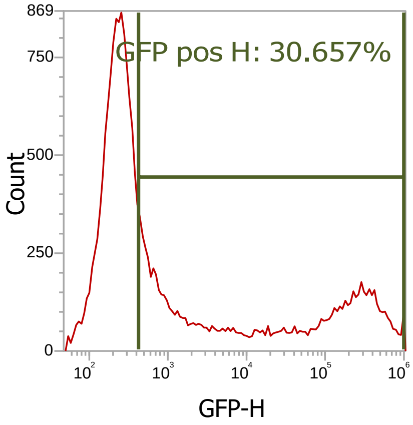

Live Cells - 11

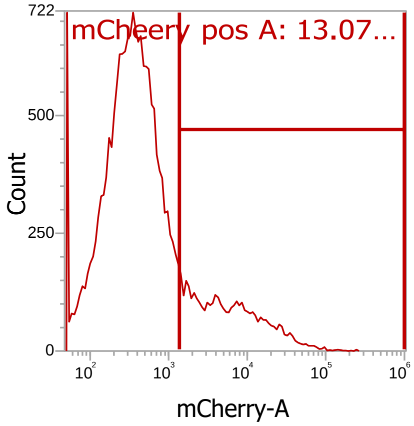

Experiment: Ljuba 22.11.2021
Sample: SINV nsP2 + VRPs
Time Recorded: 12:58:53

Name	Gate	Count	%Total	%Gated	X Mean	X Media
All Events	All Events	30,000	100.000	100.000	N/A	N/A
Live Cells	Live Cells	21,062	70.207	70.207	350,092	367,14
GFP pos H	GFP pos H	9,197	30.657	43.666	137,418	15,156
GFP pos A	GFP pos A	8,651	28.837	41.074	223,099	34,544
mCheery pos A	mCheery pos A	3,922	13.073	18.621	10,757	5,245
mCheery pos H	mCheery pos H	4,101	13.670	19.471	8,960	4,215
GFP-/mCherry+	GFP-/mCherry+	1,886	6.287	8.955	24	123
GFP+/mCherry+	GFP+/mCherry+	616	2.053	2.925	279,098	74,491
GFP-/mCherry-	GFP-/mCherry-	11,665	38.883	55.384	-8	114
GFP+/mCherry-	GFP+/mCherry-	6,895	22.983	32.737	254,857	90,047

Live Cells - 11

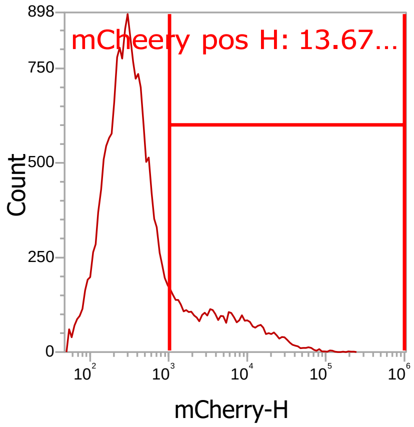

Live Cells - 11

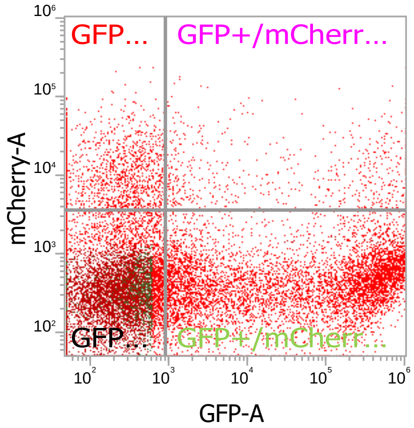

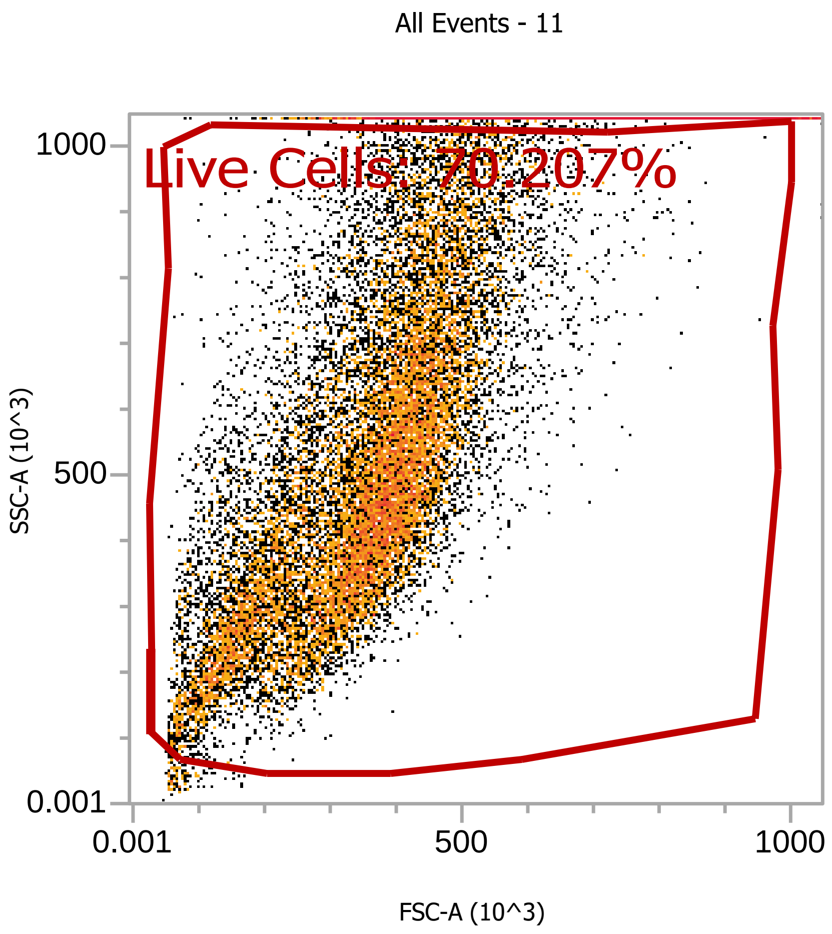
All Events - 11
Live Cells: 70.207%
1000
500
0.001
SSC-A (10^3)
0.001
500
1000
FSC-A (10^3)

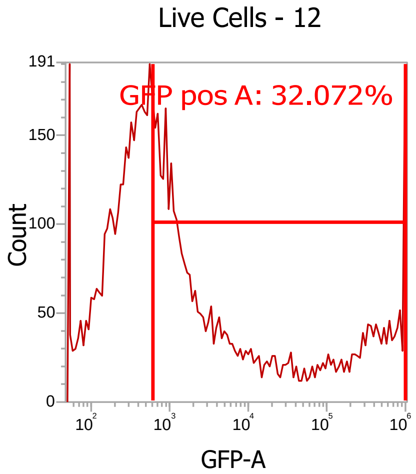

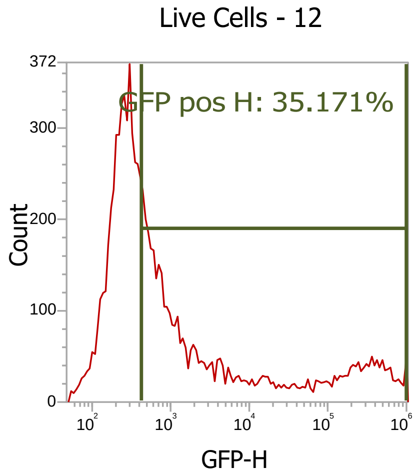

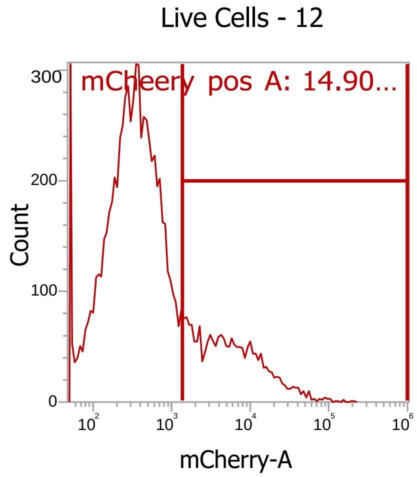

Experiment: Ljuba 22.11.2021
Sample: SINV nsP2 + VRPs
Time Recorded: 13:03:17

Name	Gate	Count	%Total	%Gated	X Mean	X Media
All Events	All Events	13,298	100.000	100.000	N/A	N/A
Live Cells	Live Cells	9,310	70.011	70.011	294,201	287,89
GFP pos H	GFP pos H	4,677	35.171	50.236	93,402	2,005
GFP pos A	GFP pos A	4,265	32.072	45.811	151,507	3,909
mCheery pos A	mCheery pos A	1,982	14.904	21.289	9,783	5,030
mCheery pos H	mCheery pos H	2,045	15.378	21.966	8,253	4,131
GFP-/mCherry+	GFP-/mCherry+	928	6.978	9.968	-39	148
GFP+/mCherry+	GFP+/mCherry+	333	2.504	3.577	236,879	15,622
GFP-/mCherry-	GFP-/mCherry-	4,938	37.133	53.040	52	219
GFP+/mCherry-	GFP+/mCherry-	3,111	23.394	33.416	182,149	9,773

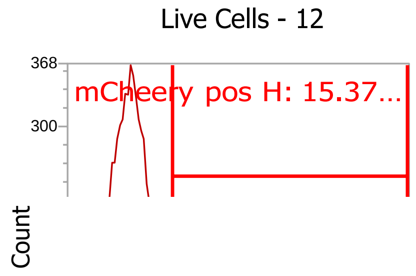

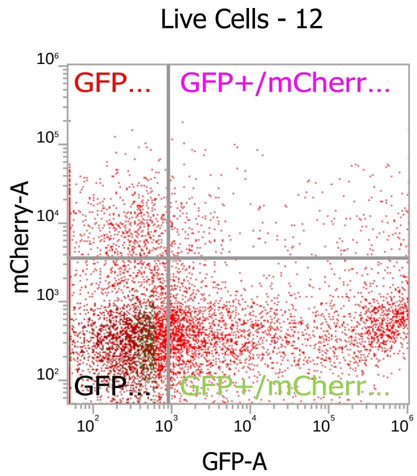

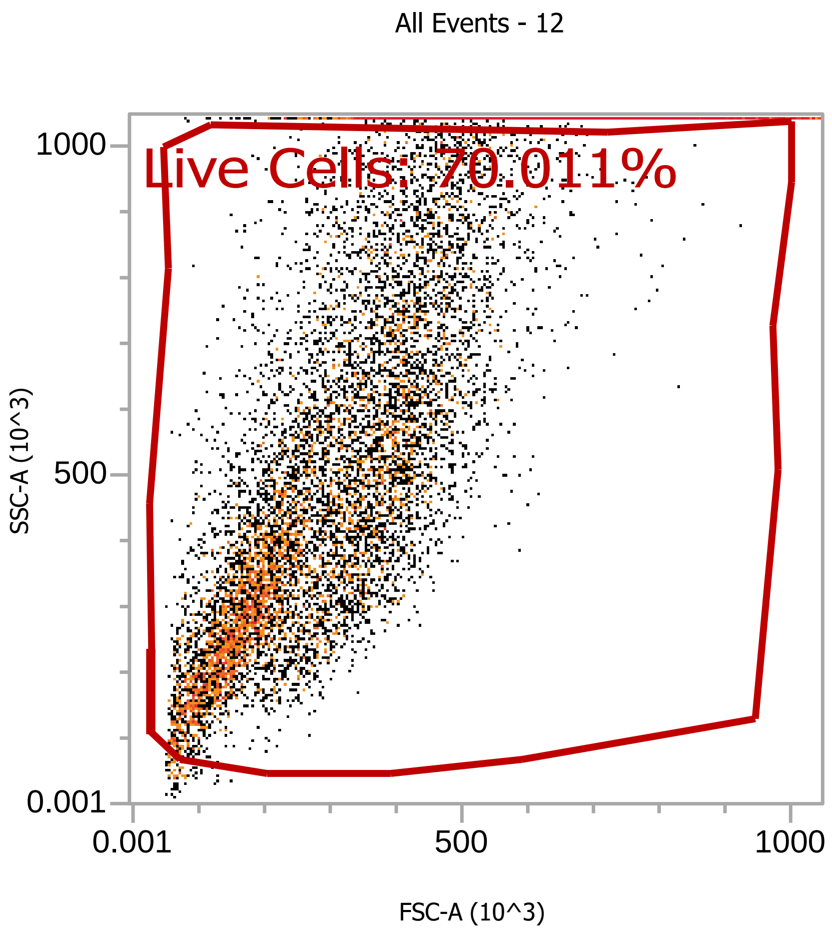
All Events - 12
Live Cells: 70.011%
1000
500
0.001
SSC-A (10^3)
0.001
500
1000
FSC-A (10^3)
