# Supplementary material for: Expression of Alphavirus Nonstructural Protein 2 (nsP2) in Mosquito Cells Inhibits Viral RNA Replication in Both a Protease Activity-Dependent and -Independent Manner
Source: Viruses. 2022 Jun 17;14(6):1327. doi: 10.3390/v14061327 (PMC9228716; doi:10.3390/v14061327)
Supplement: Supplementary file 1 [file viruses-14-01327-s001.zip › File S1 Results of FACS analysis/SINV nsP2CA infected with CHIKV VRPs.pdf]

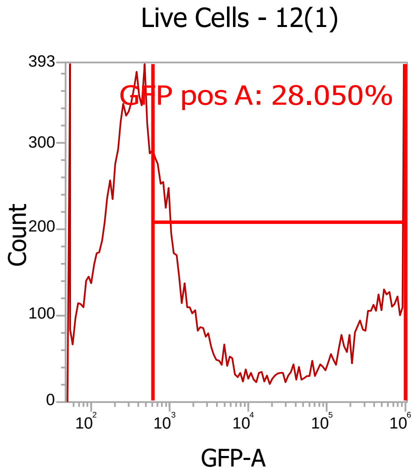

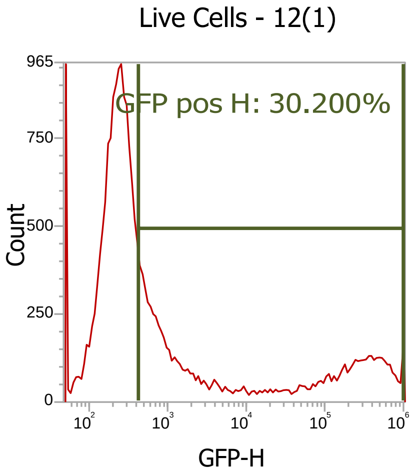

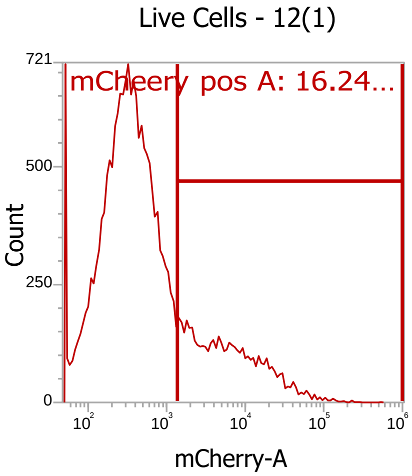

Experiment: Ljuba 22.11.2021
Sample: SINV nsP2^{CA} + VRPs
Time Recorded: 13:06:30

Name	Gate	Count	%Total	%Gated	X Mean	X Media
All Events	All Events	30,000	100.000	100.000	N/A	N/A
Live Cells	Live Cells	22,286	74.287	74.287	370,335	371,79
GFP pos H	GFP pos H	9,060	30.200	40.653	146,182	4,188
GFP pos A	GFP pos A	8,415	28.050	37.759	230,898	12,436
mCheery pos A	mCheery pos A	4,873	16.243	21.866	11,901	5,488
mCheery pos H	mCheery pos H	5,069	16.897	22.745	9,888	4,420
GFP-/mCherry+	GFP-/mCherry+	2,108	7.027	9.459	15	162
GFP+/mCherry+	GFP+/mCherry+	1,056	3.520	4.738	302,520	28,336
GFP-/mCherry-	GFP-/mCherry-	13,205	44.017	59.252	-32	119
GFP+/mCherry-	GFP+/mCherry-	5,917	19.723	26.550	274,201	84,832

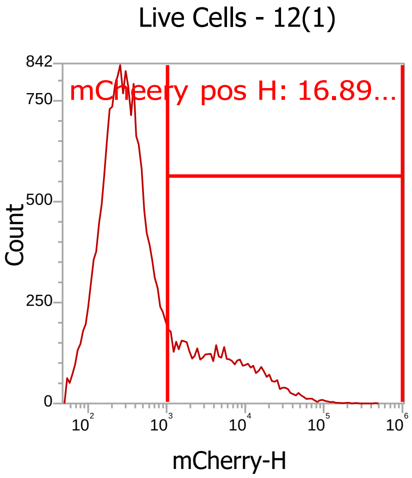

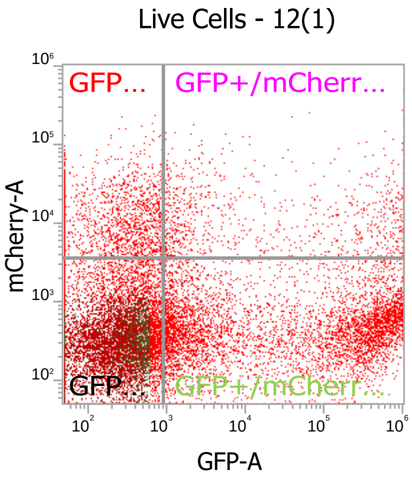

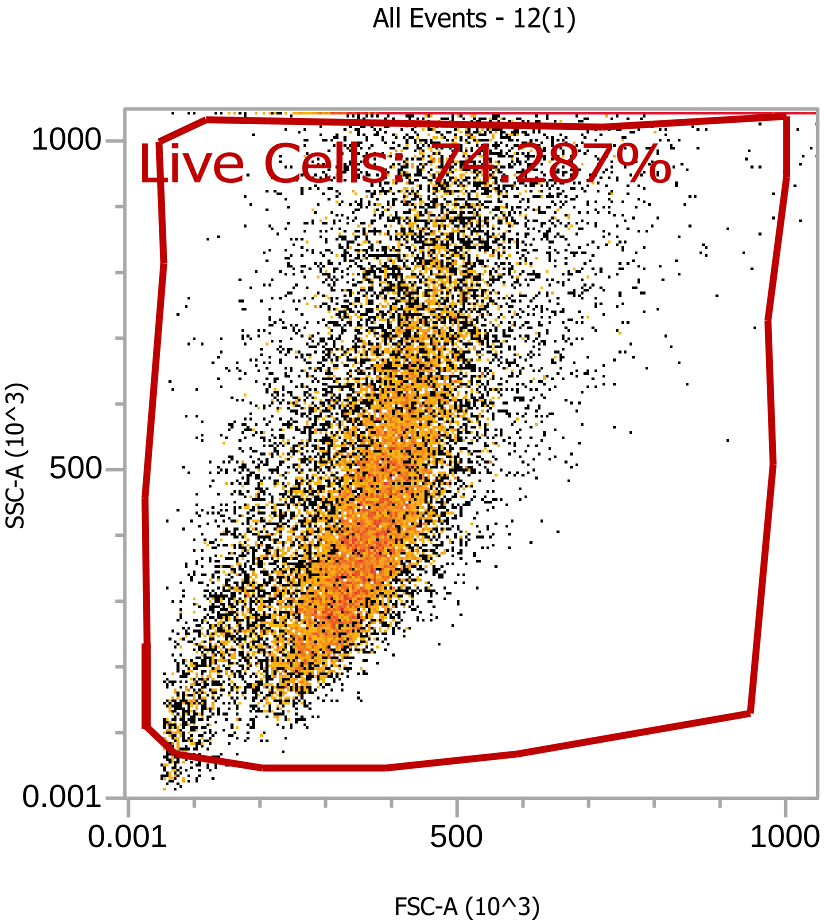
All Events - 12(1)
Live Cells: 74.287%
SSC-A (10^3)
1000
500
0.001
0.001
500
1000
FSC-A (10^3)

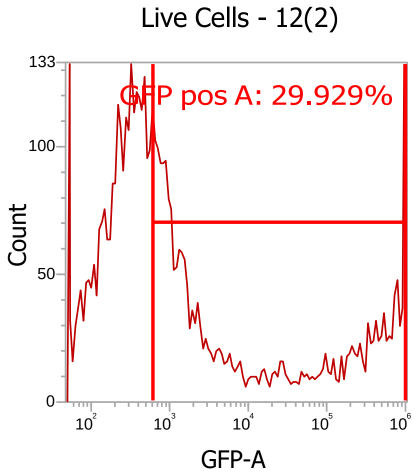

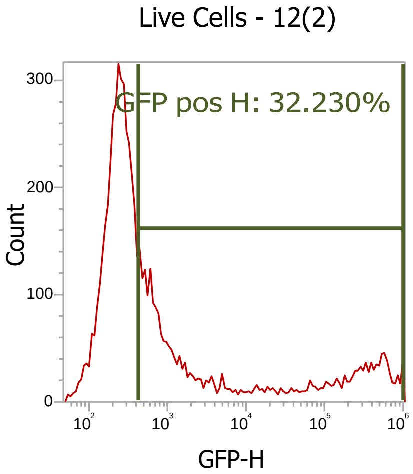

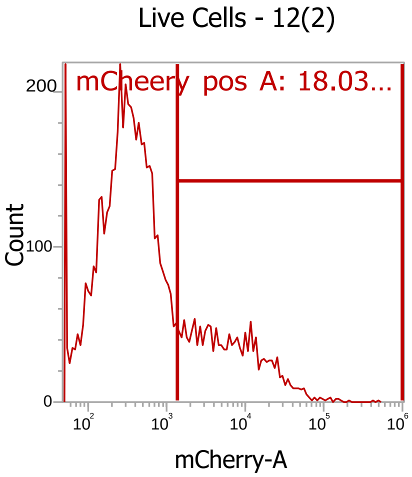

Experiment: Ljuba 22.11.2021
Sample: SINV nsP2^{CA} + VRPs
Time Recorded: 13:10:55

Name	Gate	Count	%Total	%Gated	X Mean	X Median
All Events	All Events	8,911	100.000	100.000	N/A	N/A
Live Cells	Live Cells	6,694	75.121	75.121	352,451	360,267
GFP pos H	GFP pos H	2,872	32.230	42.904	128,167	2,217
GFP pos A	GFP pos A	2,667	29.929	39.842	204,144	4,392
mCheery pos A	mCheery pos A	1,607	18.034	24.007	12,044	5,956
mCheery pos H	mCheery pos H	1,664	18.674	24.858	10,137	4,823
GFP-/mCherry+	GFP-/mCherry+	750	8.417	11.204	90	193
GFP+/mCherry+	GFP+/mCherry+	319	3.580	4.765	299,656	8,123
GFP-/mCherry-	GFP-/mCherry-	3,823	42.902	57.111	130	171
GFP+/mCherry-	GFP+/mCherry-	1,802	20.222	26.920	248,858	27,382

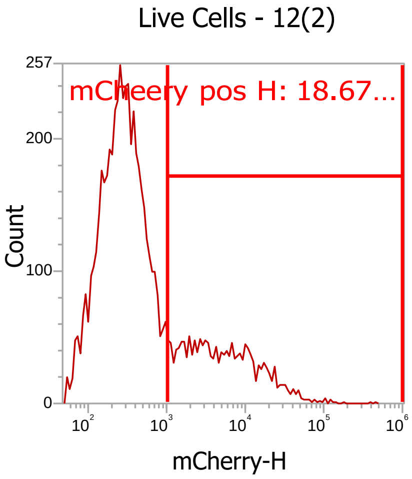

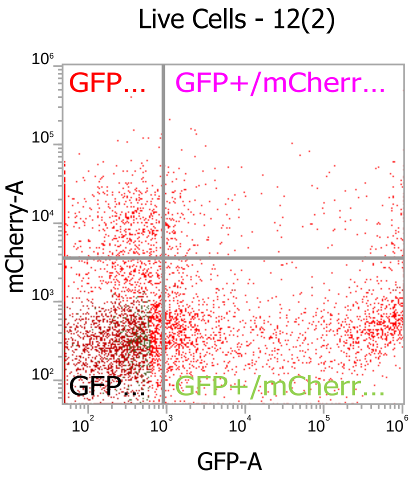

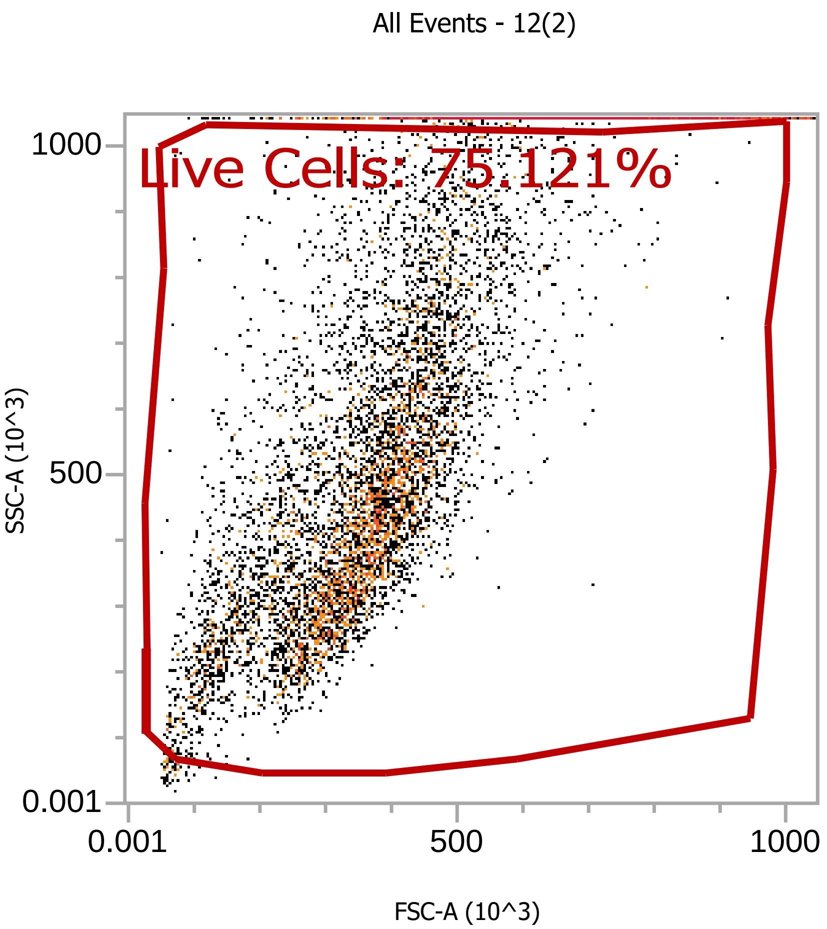
All Events - 12(2)
Live Cells: 75.121%
SSC-A (10^3)
1000
500
0.001
0.001
500
1000
FSC-A (10^3)

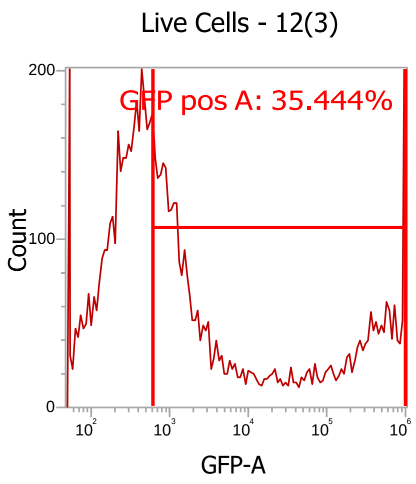

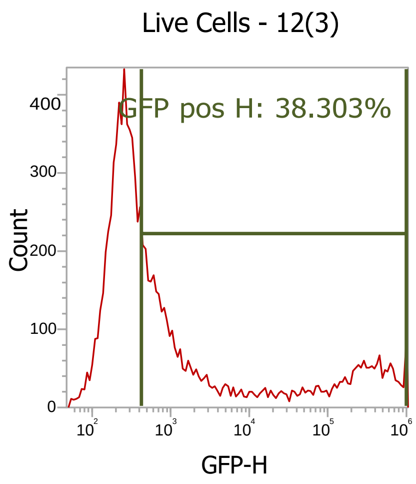

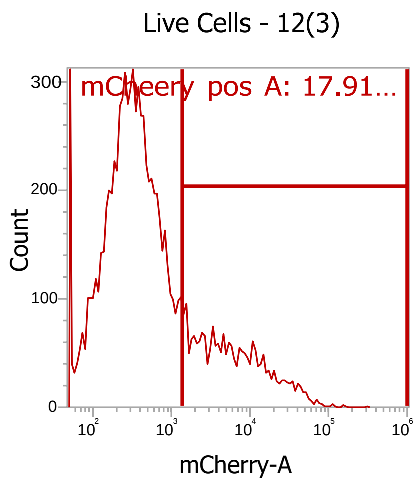

Experiment: Ljuba 22.11.2021
Sample: SINV nsP2^{CA} + VRPs
Time Recorded: 13:15:17

Name	Gate	Count	%Total	%Gated	X Mean	X Media
All Events	All Events	12,242	100.000	100.000	N/A	N/A
Live Cells	Live Cells	9,861	80.551	80.551	345,743	355,49
GFP pos H	GFP pos H	4,689	38.303	47.551	126,587	2,307
GFP pos A	GFP pos A	4,339	35.444	44.002	197,178	4,527
mCheery pos A	mCheery pos A	2,193	17.914	22.239	10,879	5,329
mCheery pos H	mCheery pos H	2,265	18.502	22.969	9,110	4,376
GFP-/mCherry+	GFP-/mCherry+	994	8.120	10.080	152	197
GFP+/mCherry+	GFP+/mCherry+	413	3.374	4.188	250,969	5,819
GFP-/mCherry-	GFP-/mCherry-	5,335	43.579	54.102	133	197
GFP+/mCherry-	GFP+/mCherry-	3,119	25.478	31.630	240,873	24,616

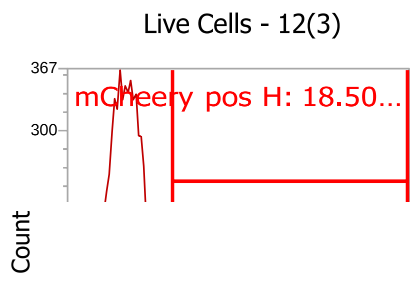

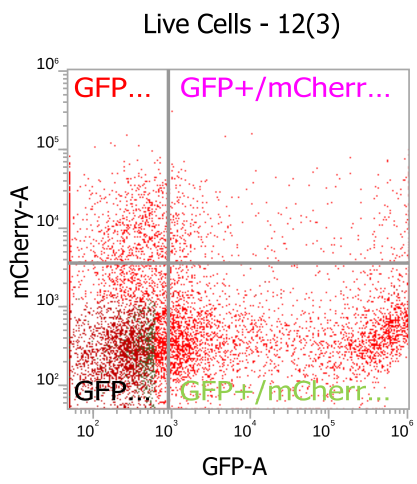

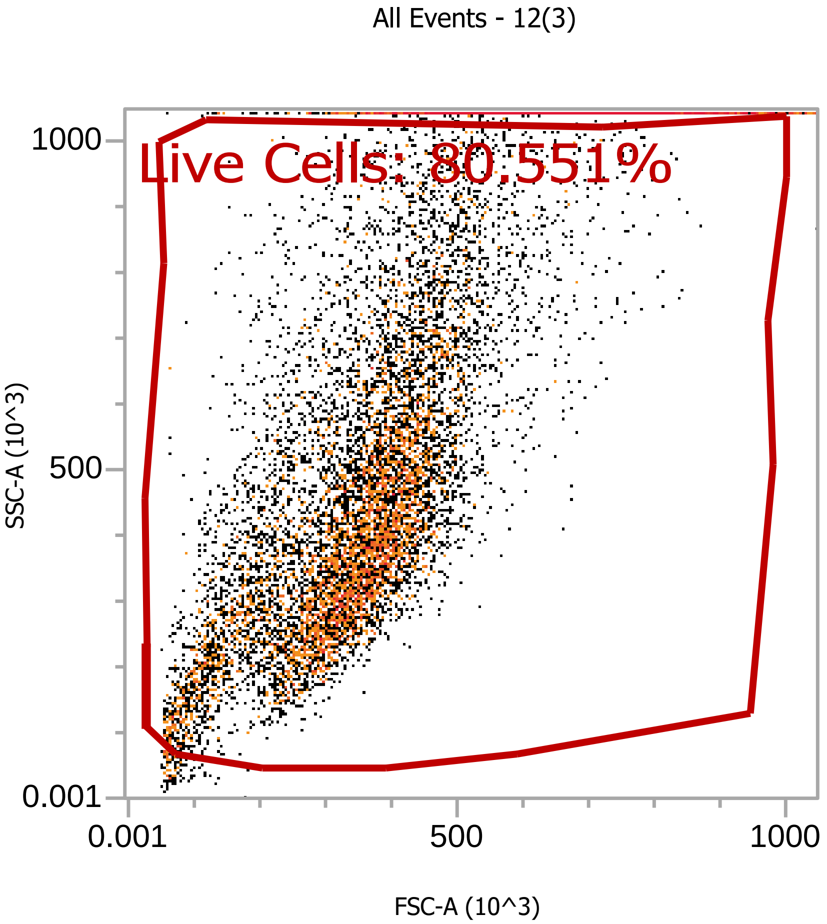
All Events - 12(3)
Live Cells: 80.551%
1000
500
0.001
SSC-A (10^3)
0.001
500
1000
FSC-A (10^3)
