# Supplementary figures and images for: Expression of Alphavirus Nonstructural Protein 2 (nsP2) in Mosquito Cells Inhibits Viral RNA Replication in Both a Protease Activity-Dependent and -Independent Manner
Source: Viruses. 2022 Jun 17;14(6):1327. doi: 10.3390/v14061327 (PMC9228716; doi:10.3390/v14061327)

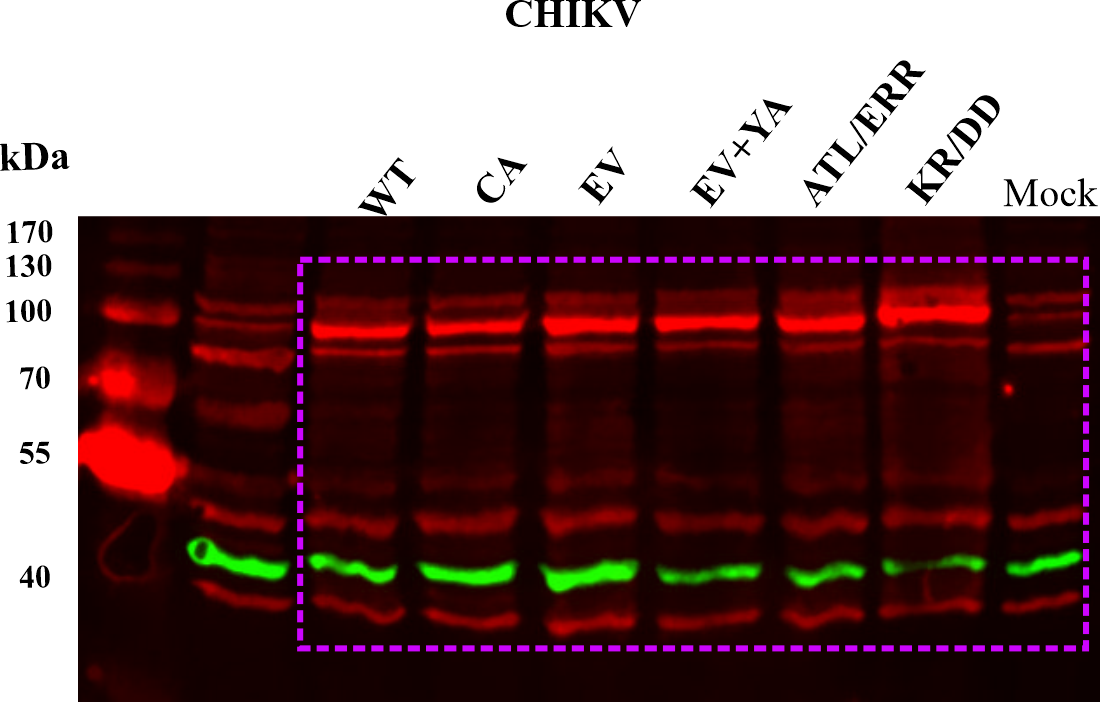

Supplement: Supplementary file 1 [file viruses-14-01327-s001.zip › File S2/CHIKV proteases.tif]

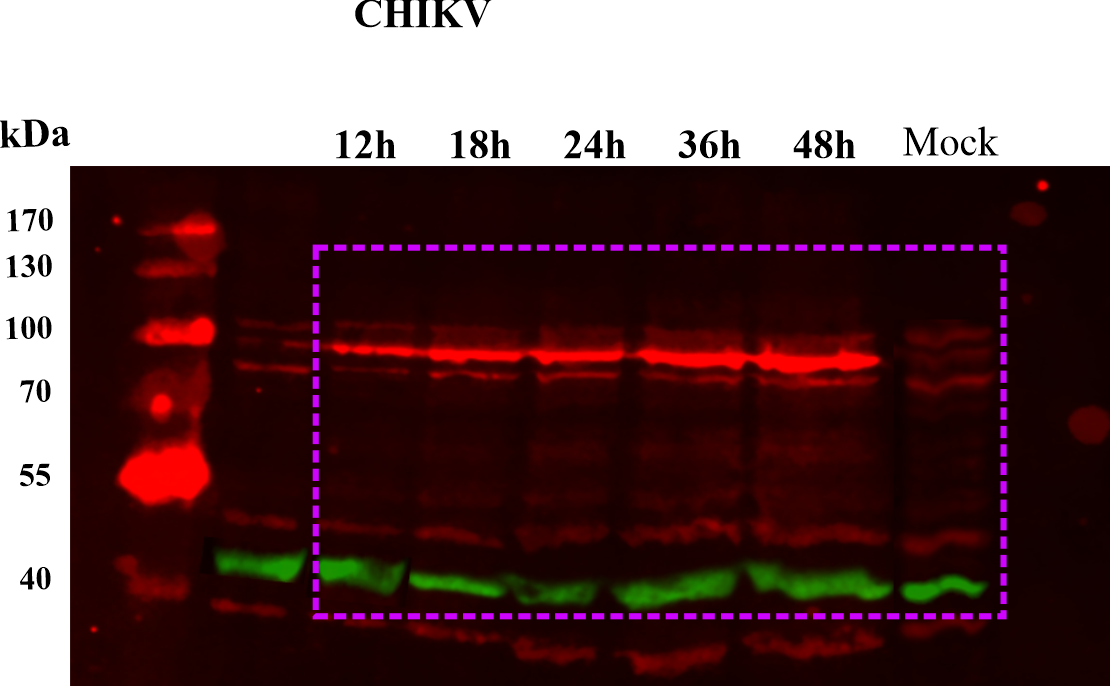

Supplement: Supplementary file 1 [file viruses-14-01327-s001.zip › File S2/CHIKV time points.tif]

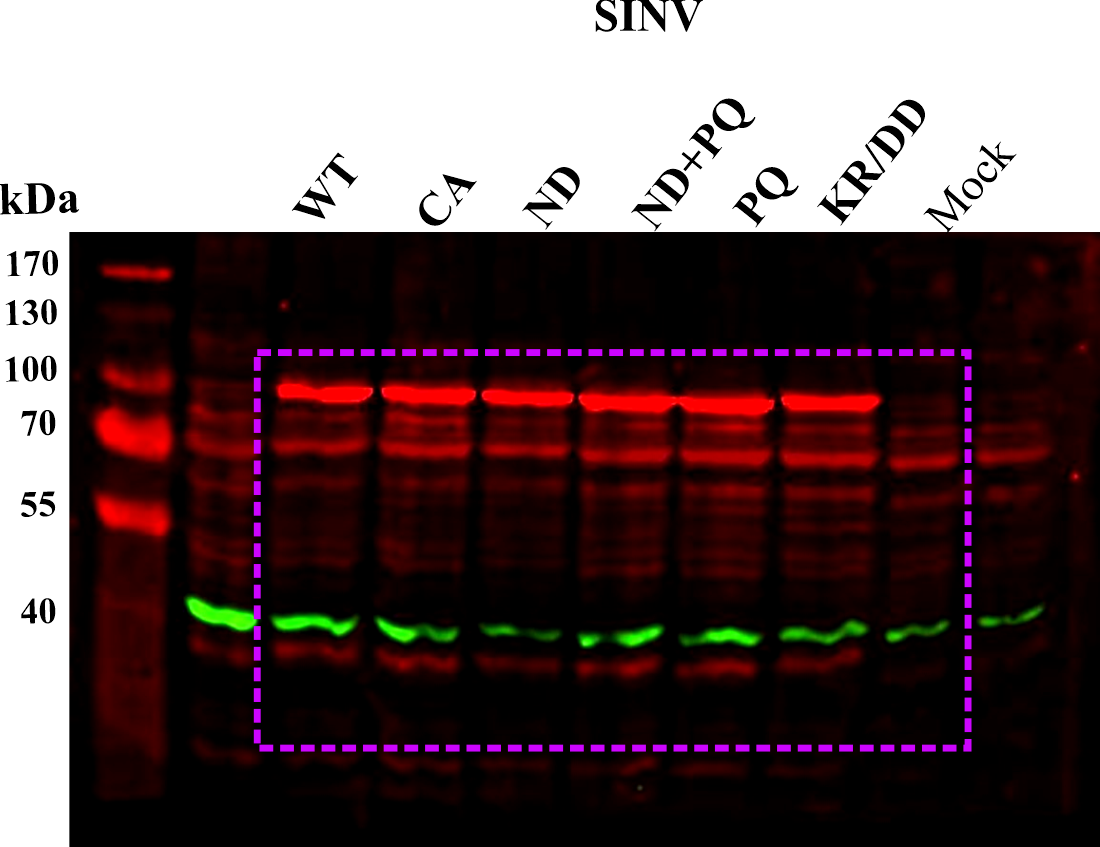

Supplement: Supplementary file 1 [file viruses-14-01327-s001.zip › File S2/SINV proteases .tif]

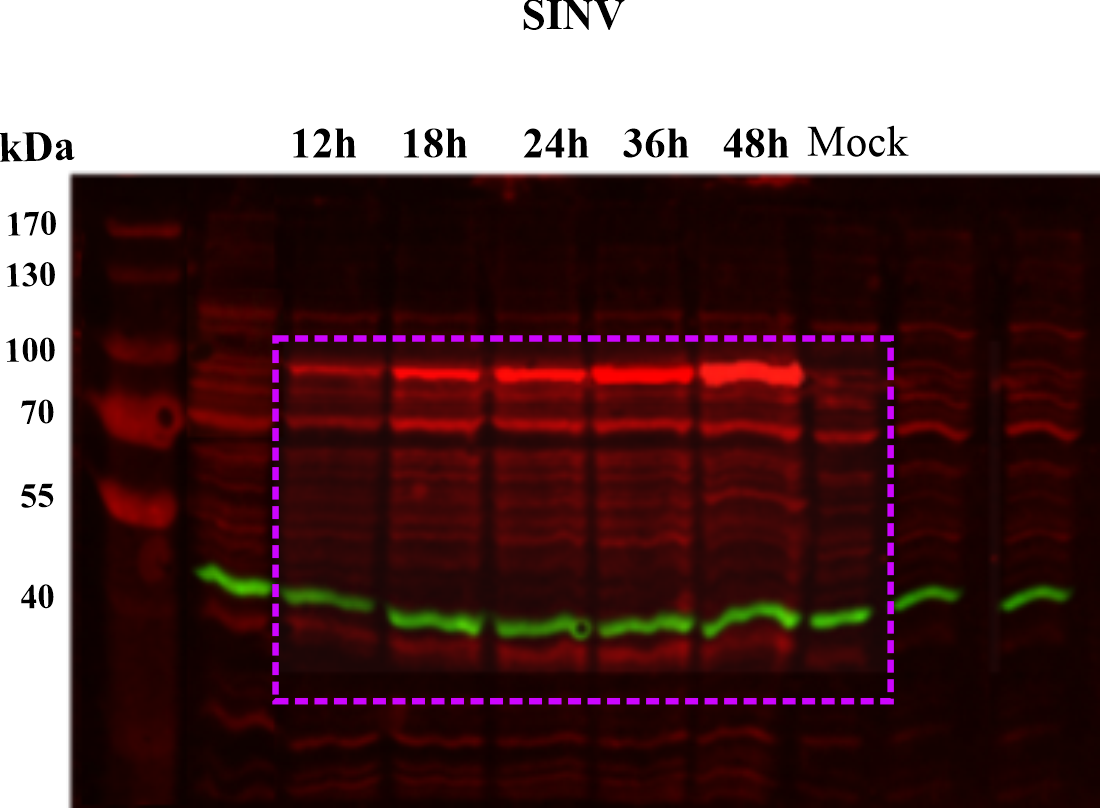

Supplement: Supplementary file 1 [file viruses-14-01327-s001.zip › File S2/SINV time points.tif]
